# Representation learning in the artificial and biological neural networks underlying sensorimotor integration

**DOI:** 10.1126/sciadv.abn0984

**Published:** 2022-06-03

**Authors:** Ahmad Suhaimi, Amos W. H. Lim, Xin Wei Chia, Chunyue Li, Hiroshi Makino

**Affiliations:** Lee Kong Chian School of Medicine, Nanyang Technological University, 11 Mandalay Road, Singapore 308232, Singapore.

## Abstract

The integration of deep learning and theories of reinforcement learning (RL) is a promising avenue to explore novel hypotheses on reward-based learning and decision-making in humans and other animals. Here, we trained deep RL agents and mice in the same sensorimotor task with high-dimensional state and action space and studied representation learning in their respective neural networks. Evaluation of thousands of neural network models with extensive hyperparameter search revealed that learning-dependent enrichment of state-value and policy representations of the task-performance-optimized deep RL agent closely resembled neural activity of the posterior parietal cortex (PPC). These representations were critical for the task performance in both systems. PPC neurons also exhibited representations of the internally defined subgoal, a feature of deep RL algorithms postulated to improve sample efficiency. Such striking resemblance between the artificial and biological networks and their functional convergence in sensorimotor integration offers new opportunities to better understand respective intelligent systems.

## Teaser

Representational convergence was identified between the brain and machine underlying sensorimotor integration.

## INTRODUCTION

In reinforcement learning (RL), biological and artificial agents learn to optimize their actions to maximize future cumulative rewards in response to sensory inputs derived from each state of the environment. As RL is closely related to reward-based learning and decision-making in humans and other animals, it has provided theoretical frameworks for neuroscience (*1*–*6*) and neural representations of various RL-related decision variables have been identified (*7*–*23*).

Deep learning has offered valuable research tools to model brain functions under the scheme of supervised learning, where the “correct” answers are provided during training. Deep learning adjusts weights in the neural network to establish a desired input-out mapping via nonlinear function approximation, which permits generalization across different inputs. A performance-optimized deep neural network mapping sensory inputs on neural activity, for example, successfully predicted neural activity in the visual system at unprecedented accuracy (*24*). Thus, the deep neural network has successfully served as an outstanding model of neural representation in neuroscience (*25*–*27*).

The recent emergence of deep RL has exploited theoretical frameworks of learning and decision-making of RL and the representation power of deep learning to express the flow of information from perception to action in an “end-to-end” manner (*28*, *29*). As the brain shares a similar network, deep RL algorithms are considered to offer new hypotheses on decision-making in the brain (*30*). Recent studies have demonstrated potential benefits of deep RL to model reward-based learning and underlying neural representations (*31*–*33*).

Learning in machines is considerably slow and data-hungry compared to humans and other animals, and new algorithms have been continuously developed in deep RL to improve sample efficiency. For example, discovery of the “subgoal” by assigning an intrinsic value is useful for the artificial agent to improve learning by decomposing a task into simpler subproblems (*34*, *35*). Such task decomposition facilitates skill transfer to other tasks where the same subgoal is useful (*36*). However, whether analogous algorithms are used in the brain has not been extensively explored.

To identify commonalities and discrepancies in operation principles between the artificially and biologically intelligent systems, we performed comparative analysis between the deep RL agent and mouse cortex and studied representation learning in their respective neural networks underlying the same sensorimotor task. By leveraging on the use of a two-photon random access mesoscope (2p-RAM) (*37*), we imaged thousands of neurons across individual cortical regions. Among the six regions we examined, the mouse posterior parietal cortex (PPC) shared similar representations with the artificial neural network (ANN) of the task-performance-optimized artificial agent trained with a deep RL algorithm. Our results highlight marked resemblance between the PPC and ANN of the deep RL agent in representation learning underlying sensorimotor integration, and provide new insights into how the efficient computation of the brain can be implemented in the machine.

## RESULTS

### Sensorimotor task for the mouse

**Fig. 1.**
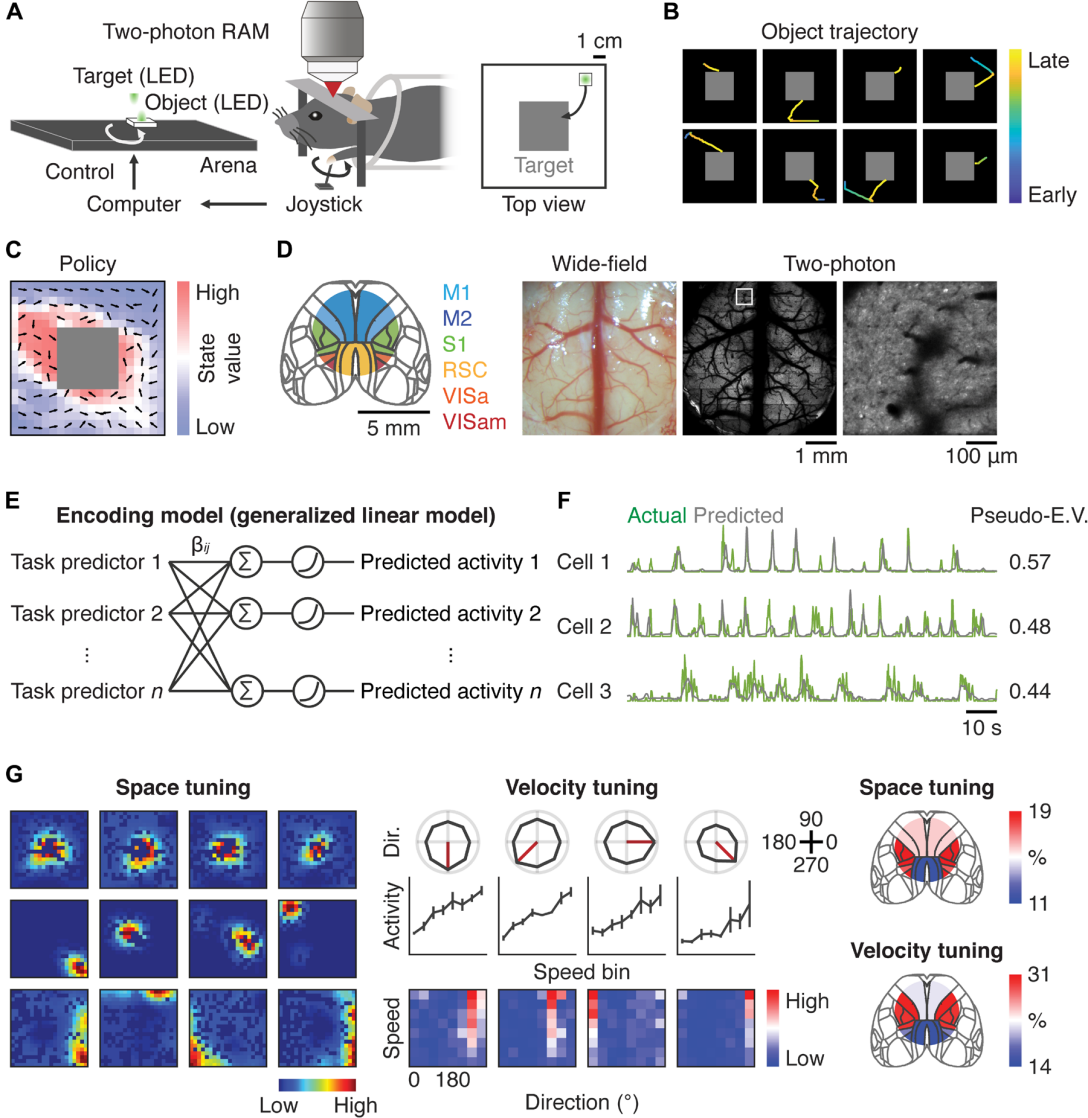
Space and velocity representations of a manipulated object in the mouse cortex. (**A**) Schematic of the object manipulation task for the head-restrained mouse. RAM, random access mesoscope. The gray square indicates the reward zone. (**B**) Example object trajectories of eight consecutive trials for an expert mouse. Color represents time. Median trial duration across mice was 2.1 ± 0.3 s (median ± SEM; *n* = 9 mice). (**C**) Example of the state-value function indicated by the heatmap and policy denoted by the unit vectors. (**D**) Six imaged regions of the mouse cortex and example images of the cortex under wide-field and two-photon microscopes. Right panel in two-photon is a zoomed image indicated by the white box. M1, primary motor cortex; M2, secondary motor cortex; S1, primary somatosensory cortex; RSC, retrosplenial cortex; VISa, anterior visual cortex; VISam, anteromedial visual cortex. VISa and VISam were considered collectively as the posterior parietal cortex (PPC). (**E**) Generalized linear model (GLM) for neural activity based on task predictors. The task predictors were obtained by convolution of task variables with basis functions in space or time. β*_ij_* is a weight of each task predictor. (**F**) Example neurons with their actual and modeled activity by GLM. Pseudo-E.V., pseudo-explained variance. (**G**) Left: Examples of space-tuned neurons for the object. Middle: Examples of velocity-tuned neurons for the object. The speed bins are eight uniformly spaced numbers between 1.4 and 21.3 cm/s. Red lines indicate the preferred direction of each neuron. For space- and velocity-tuned neurons, each neuron was statistically identified with a permutation test (*P* < 0.05). Right: Cortical distribution of space- or velocity-tuned neurons.

We designed an object manipulation task involving a high-dimensional state and action space for water-restricted and head-restrained mice. They were trained to use a joystick to remotely manipulate an object with a light-emitting diode (LED) in a 10 cm × 10 cm arena toward a reward zone located in the center (4 cm × 4 cm), indicated by a target LED (Fig. 1A). At each trial onset, the object was positioned at a random location outside the reward zone, and both object and target LEDs were turned on. The trials ended when mice successfully moved the object toward the reward zone, where the object was made stationary, and received a drop of water (8 μl), or when 5 min elapsed. Mice gradually became proficient at this task and reached a hit rate of 99.95 ± 0.03% (mean ± SEM; *n* = 9 mice), with significant decreases in the trial duration and object’s distance traveled at the expert stage (Fig. 1B and fig. S1, A and B). Expert mice did not just randomly move the joystick but used visual feedback to guide their actions (fig. S1, C to E). These results demonstrate that head-restrained mice successfully learned to remotely control the object using a joystick.

In computational RL, the state-value function, denoted as *V*^π^(*s*), estimates the expected sum of future rewards in each state *s* when the agent follows the policy (action probability in each state) π thereafter (*2*). To assign a value for each state of the arena, we defined *V*^π^(*s*) as

$$V^{\pi }(s_{t})=E[R_{t}+\gamma R_{t+1}+\gamma^{2}R_{t+2}+\cdots +\gamma^{T-t}R_{T}]$$ (1)

where *s_t_* corresponds to the location of the object in the arena at time *t*, E denotes expectation, *R_t_* is a reward at time *t*, γ is a discount factor, and *T* is a trial end. Because in our task the mouse did not receive a reward of 1 elsewhere other than the reward zone, *V*^π^(*s*) can be simplified as (*38*)

$$V^{\pi }(s_{t})=E[\gamma^{T-t}]$$ (2)

Thus, *V*^π^(*s*) is equivalent to the monotonic measure of the mean time to reach the reward zone from each state. π was defined as the probability of the object movement direction for a given state *s*. Over learning, *V*^π^(*s*) increased throughout different states and π changed so that the preferred object movement was more directed toward the reward zone, indicating that the behavior for the task was gradually optimized (Fig. 1C and fig. S1F). These results establish that the object manipulation task is able to derive computational RL variables such as *V*^π^(*s*) and π.

### Space and velocity tuning of the object in the mouse cortex

We next studied neural representations of task variables in the mouse cortex by imaging activity of excitatory neurons in transgenic mice (CaMKII-tTA×TRE-GCaMP6s) using a 2p-RAM (Fig. 1D). Calcium imaging with 2p-RAM permits simultaneous activity recordings from thousands of neurons across distant cortical regions with cellular resolution. Our imaging window included six cortical regions: the primary motor cortex (M1), secondary motor cortex (M2), primary somatosensory cortex (S1), retrosplenial cortex (RSC), anterior visual cortex (VISa), and anteromedial visual cortex (VISam) (Fig. 1D). We considered VISa and VISam to be collectively corresponding to the PPC while acknowledging that there exists mixed conceptual agreement in the literature (*39*).

To determine task representations in each neuron, we built an encoding model [generalized linear model (GLM)], which incorporated measured task variables as predictors to model neural activity (Fig. 1E and fig. S2A) (*40*, *41*). The task variables included in the model were the trial onset and offset times, object velocity, object position, joystick velocity, and reward onset times. We assessed each model performance quantitatively by computing pseudo-explained variance (E.V.) on the data that were held out from the model fitting procedure (Fig. 1F) (*42*). We detected 48 ± 4% (mean ± SEM; *n* = 9 animals) of analyzed neurons that were significantly modulated by task variables and defined these cells as task-related neurons.

To quantify the relative contribution of each task variable to the activity of individual task-related cells, we excluded one task variable from the model and tested whether the performance of the resulting partial model degraded relative to the full model (fig. S2B). A task variable was deemed to be contributing to the activity when the full model was quantitatively better than the partial model based on the pseudo-E.V. (fig. S2, C and D) (*42*). Overall, 74 ± 2% (mean ± SEM; *n* = 9 mice) of task-related neurons showed mixed selectivity (*43*), whose activity was modulated by some combination of the task variables (fig. S2E).

By factoring the multiplexed representations, we computed in each neuron a model-derived response profile for a given task variable, which is analogous to a tuning curve (*44*). Activity analysis of cortical neurons with reference to the object position revealed their space tuning (Fig. 1G). Space-tuned neurons had a “place field” or were preferentially activated at the border of the arena (Fig. 1G and fig. S2F). Such model-derived space tuning was robust across three calcium signal deconvolution methods [Suite2p (*45*), MLspike (*46*), and LZero (*47*); fig. S2G]. The space-tuned neurons were enriched in areas such as S1 and PPC (Fig. 1G and fig. S2H). Activity of cortical neurons was also selectively modulated by the velocity of the object movement such that the speed modulation was prominent for the preferred direction (Fig. 1G). The velocity-encoding neurons were enriched in regions such as M1 and S1 (Fig. 1G and fig. S2H).

### Evaluation of thousands of deep RL agent models

**Fig. 2.**
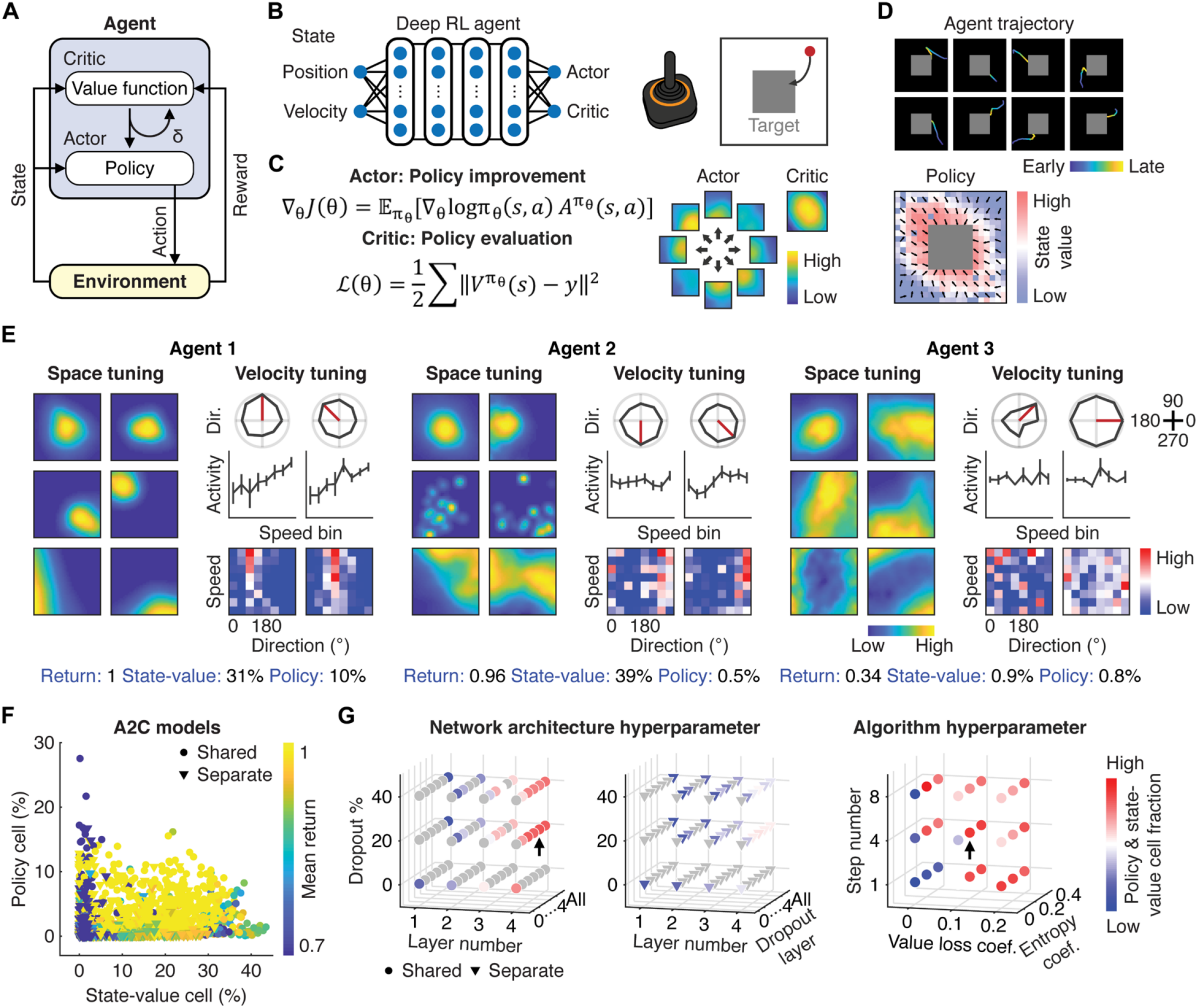
Performance optimization and neural representations in deep RL agent models. (**A**) Schematic of the actor-critic architecture of the deep RL agent interacting with the environment. δ denotes a temporal difference error. (**B**) Schematic of the ANN of the deep RL agent and the task. The gray square indicates the reward zone. (**C**) Left: The actor and critic correspond to policy improvement and policy evaluation, respectively. Right: Outputs of the actor and critic in the space coordinate. Note that the actor outputs were activated in conjunction with states (heatmap) and actions (arrows), while the critic output depended on states only. (**D**) Top: Example trajectories of eight consecutive trials for a trained deep RL agent. Bottom: Example of the state-value function indicated by the heatmap and the policy denoted by the unit vectors. (**E**) Examples of space and velocity tuning of neurons in different RL agent models. Agent 1, task-performance-optimized RL model. Agents 2 and 3, suboptimal (mean return below 1) RL models. (**F**) Relationship between the task performance and the state-value- and policy-encoding cell fractions in deep RL agent models trained with the Advantage Actor-Critic (A2C). Each point represents an RL agent model (*n* = 1620 models, four runs averaged per model). Circles and triangles indicate whether the actor and critic networks were shared or separated, respectively. Color denotes the mean return, a metric used to evaluate the task performance of the model. Note that representations of the policy and state-value were competed when the networks were shared. (**G**) Policy- and state-value-encoding cell fractions in the hyperparameter space for the network architecture and algorithm. The arrows are the selected model parameters for further analysis.

To compare representation learning between artificially and biologically intelligent systems, we built the same task environment and trained artificial agents with a deep RL algorithm known as the Advantage Actor-Critic (A2C) (Fig. 2, A and B) (*48*). Because the movement of mice was constrained due to the relative position of the joystick (fig. S1A), we modified the RL agent’s action distribution to mimic the object movement made by mice. A2C is a model-free RL algorithm where the policy gradient and value-based methods are combined. The actor-critic algorithm was selected as, unlike other algorithms, it entails representations for both state-value estimation (critic) and policy computation (actor) in their ANN, which are critical for decision-making in the brain. The objective of the policy gradient algorithm *J*(θ), which is used in the actor, is the expected sum of future rewards

$$J(\theta )=E_{\pi_{\theta }}[\sum_{t=1}^{T}R(s_{t},a_{t})]$$ (3)

where θ is the weight of the neural network, *R* is a reward, and *a_t_* is an action at time *t*. The actor updates policy parameters θ by performing gradient ascent of this objective according to the policy gradient theorem

$$\nabla_{\theta }J(\theta )=E_{\pi_{\theta }}[\nabla_{\theta }\text{log}\pi_{\theta }(s,a)\;A^{\pi_{\theta }}(s,a)]$$ (4)

where ∇_θ_logπ_θ_(*s*, *a*) is the gradient of log probability of taking an action *a* given a state *s*, and *A*^π_θ_^(*s*, *a*) is the advantage function describing the value of a particular action relative to the average. Intuitively, the actor increases the probability of good actions and lowers the probability of bad actions based on value estimates (policy improvement). The critic on the other hand learns to estimate *V*^π_θ_^(*s*) through supervised regression via function approximation with the neural network (policy evaluation)

$$ℒ(\theta )=\frac{1}{2}\sum {\left\|V^{\pi_{\theta }}(s)-y\right\|}^{2}$$ (5)

where ℒ(θ) is a loss function and *y* is a target value, such as a bootstrapped estimate using the previously fitted *V*^π_θ_^(*s*). *V*^π_θ_^(*s*) is then used to estimate the advantage function *A*^π_θ_^(*s*, *a*) to compute the policy gradient.

We trained thousands of deep RL agents by exploring the hyperparameter space for the network architecture and the algorithm. The network architecture hyperparameters included whether the actor and critic were shared in the same network or separated in independent networks, the number of layers, the location of the dropout layer, and the fraction of dropout, while the algorithm hyperparameters included the coefficient of value-function loss, the coefficient of policy entropy, and the number of future steps to be considered to estimate *V*^π^(*s*). We confirmed that outputs of the actor and critic network corresponded to the agent’s policy and state-value, respectively (Fig. 2C). For example, the actor output for an upward action of the agent was high when the agent was located at the bottom of the arena. During training, *V*^π^(*s*) and π of the deep RL agent whose task performance was optimized improved qualitatively similarly to those of the object controlled by mice (Figs. 1C and 2D and fig. S1, F and G).

We next examined post-training activity of ANNs in thousands of artificial RL agent models. After training, we observed the emergence of space and velocity tuning in neurons of the hidden layers of the ANN (Fig. 2E). Spatial coherence of space-tuned neurons and the fraction of velocity-tuned neurons were positively correlated with the performance of the agents (fig. S3A). In other words, when the agent’s task performance was optimized, the space and velocity tuning of neurons in the ANN became analogous to those observed in the mouse cortex. This was not the case in suboptimal agents with a mean return of less than one, which had lower fractions of the state-value- and policy-encoding neurons with reduced spatial coherence and a lower fraction of velocity-tuned cells (Fig. 2E and fig. S3A).

To test whether the space tuning in the deep RL agent corresponded to the state-value representation, we computed Pearson’s correlation coefficient between the space tuning of individual neurons with *V*^π^(*s*). For the policy representation, because its outputs depended on state-action pairs, we examined each neuron’s space and direction tuning. We determined the action distribution of the agent at the spatial bins with high activity and compared this distribution with the direction tuning of the cell by measuring Pearson’s correlation coefficient. Neurons were classified either as state-value- or policy-representing when each metric was statistically higher than the one computed by chance.

Exploration of the hyperparameter space revealed a positive relationship between agents’ task performance and the fractions of policy- and state-value-representing neurons in their ANNs such that both kinds of neurons had to be abundant for the agent to perform well (Fig. 2F). The fractions of these state-value- and policy-encoding neurons were high when their networks were shared and deep (Fig. 2G and fig. S3B). Such network architecture resembled the mouse cortex where representations of task variables were shared at the level of individual neurons (fig. S2E). Moreover, high degrees of coefficients for the value-function loss and policy entropy were also important to maximize the fraction of these neurons (Fig. 2G and fig. S3B). These results suggest that potential constraints related to these hyperparameters were imposed in the mouse cortex to express the observed tuning properties. Hereafter, we focus on the task-performance-optimized deep RL agent model for further analysis.

### State-value and policy representations in the deep RL agent and mouse cortex

**Fig. 3.**
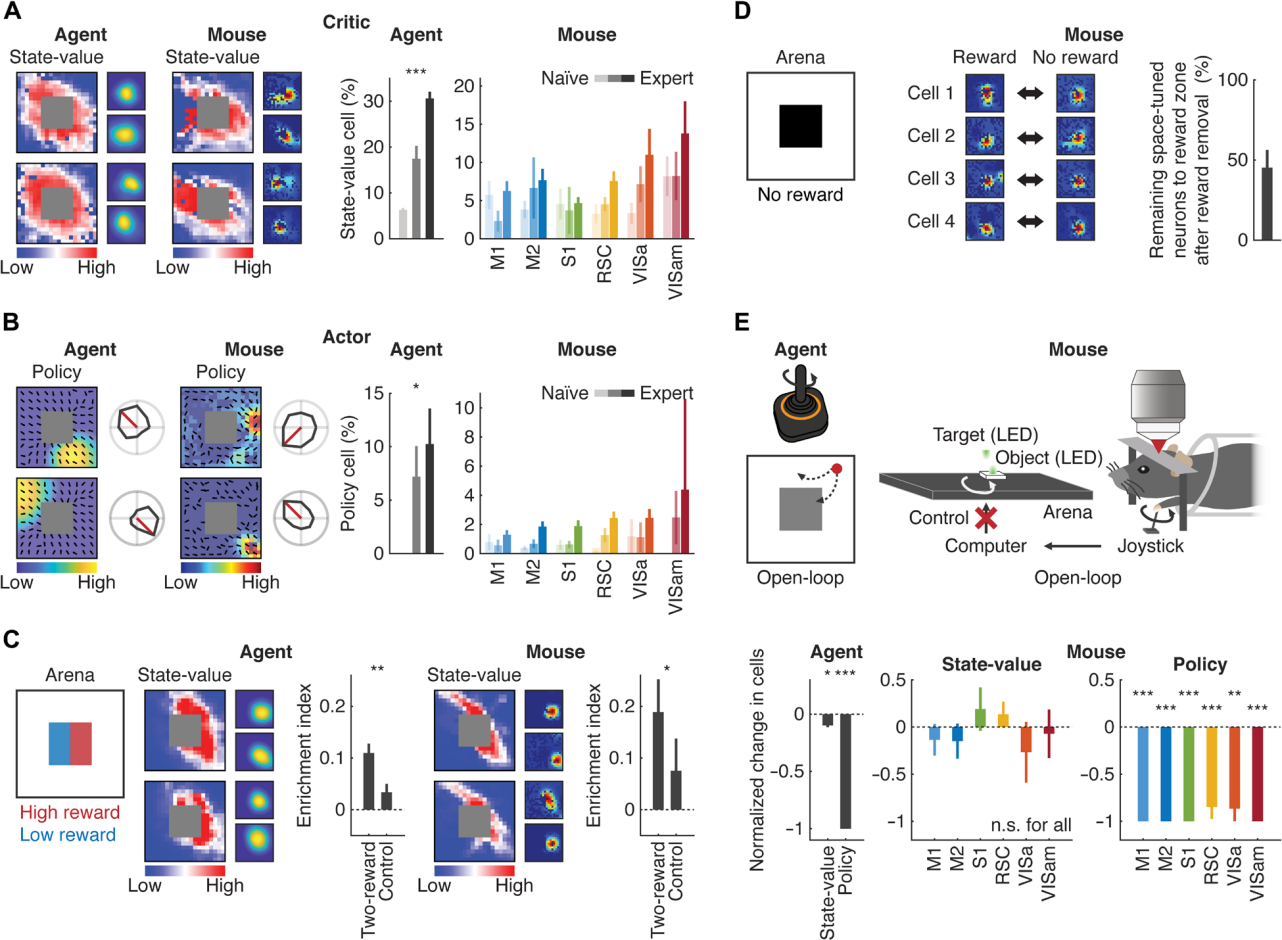
State-value and policy representations in the deep RL agent and mouse cortex. (**A**) Left: Examples of the state-value function and space tuning of state-value neurons in the deep RL agent and mouse cortex. Right: Learning-dependent increase in the fraction of state-value neurons in the deep RL agent [***P < 0.001, one-way analysis of variance (ANOVA); *n* = 4 agents] and different cortical regions. (**B**) Left: Examples of the space (left) and direction (right) tuning of policy neurons in the deep RL agent and mouse cortex. Right: Same as (A) for policy neurons (*P < 0.05, one-way ANOVA; *n* = 4 agents). (**C**) Left: Schematic of the two-reward-magnitude experiment. Middle: Examples of the state-value function and space tuning of neurons in the deep RL agent. Enrichment index was computed on the basis of the peak location of each space-tuned neuron in the two-reward (e.g., right versus left) and control (e.g., bottom versus top) side comparisons (**P < 0.01, *t* test; *n* = 8 agents). Right: Same for the mouse cortex (*P < 0.05, *t* test; *n* = 9 mice). (**D**) Left: Schematic of the interleaved-reward experiment. Middle: Examples of reward zone tuning with and without the reward. Right: Fraction of the reward zone–tuned neurons remaining after reward removal. (**E**) Top: Schematic of the open-loop experiment in the deep RL agent (left) and mouse (right). Bottom: Normalized change in the fraction of state-value and policy neurons from the closed-loop to open-loop configuration in the deep RL agent (left: *P < 0.05 and ***P < 0.001, *t* test with Bonferroni correction; *n* = 4 agents) and each cortical region (right: ***P < 0.001 and **P < 0.01, bootstrap with Bonferroni correction; *n* = 9 and 7 mice for closed-loop and open-loop). n.s., not significant.

In the deep RL agent, the state-value- and policy-representing neurons became enriched over the course of learning (*P* < 0.001; *n* = 4 agents; Fig. 3, A and B). We hypothesized that the space and velocity tuning of neurons in the mouse cortex were also linked to state-value and policy representations. To evaluate state-value representations in the mouse cortex, we computed Pearson’s correlation coefficient between the space tuning of individual neurons with *V*^π^(*s*) of the mouse (Fig. 3A). Overall, 22.9% of space-tuned neurons (*n* = 5273 cells) were statistically (*P* < 0.05) deemed to be encoding *V*^π^(*s*) (expected percentage by chance assuming uniform distribution of space tuning across the arena: 17.9%; *P* < 0.001). Although these neurons were widely distributed across different cortical areas, they were abundant in the PPC (VISa: *P* = 0.08, 0.14, 0.03, and 0.09; VISam: *P* = 0.008, 0.06, 0.005, and 0.02 compared with M1, M2, S1, and RSC, respectively; *n* = 9 mice, bootstrap), and the enrichment depended on learning in a manner similar to the deep RL agent (M1: *P* = 0.44; M2: *P* = 0.03; S1: *P* = 0.44; RSC: *P* = 0.01; VISa: *P* = 0.02; VISam: *P* = 0.12, naïve versus expert; *n* = 6 and 9 mice for naïve and expert, bootstrap; Fig. 3A and fig. S2I).

To study whether there exist policy representations in the mouse cortex, we discretized object movement into eight actions (0°, 45°, 90°, 135°, 180°, 225°, 270°, or 315°). We focused on neurons exhibiting conjunctive coding for object position and velocity as the policy depends on a state (space tuning) and action (velocity tuning) pair (19.0% conjunctive among neurons that were tuned to either the object position or object velocity). Overall, 17.4% of neurons (*n* = 2090 cells) with conjunctive coding properties were statistically (*P* < 0.05) encoding the policy (expected percentage by chance assuming uniform distribution of space tuning: 5.6%; *P* < 0.001). Distribution of the policy-representing neurons was also enriched in the PPC (VISa: *P* = 0.02, 0.16, 0.18, and 0.48; VISam: *P* = 0.003, 0.006, 0.006, and 0.03 compared with M1, M2, S1, and RSC, respectively; *n* = 9 mice, bootstrap), and the enrichment depended on learning (M1: *P* = 0.23; M2: *P* = 0.002; S1: *P* = 0.01; RSC: *P* = 0; VISa: *P* = 0.13; VISam: *P* = 0, naïve versus expert; *n* = 6 and 9 mice for naïve and expert, bootstrap; Fig. 3B and fig. S2I). Such learning-dependent enrichment of the state-value and policy representations in both the deep RL agent and mouse cortex suggests their important contribution to decision-making during the task.

To further confirm that these neurons represented the state-value and policy, we performed three sets of experiments. First, we manipulated *V*^π^(*s*) by splitting the reward zone into half. The mouse received a higher reward (10 μl) when the object reached one-half of the reward zone and a lower reward (1 μl) when it reached the other half of the reward zone. Second, we omitted the reward in 50% of randomly interleaved trials to test whether neural representations do not simply reflect the reward per se. Third, we performed an open-loop experiment where the joystick was decoupled from the object movement while the object was moved randomly.

When the reward magnitude was altered, space representations of neurons in the deep RL agent shifted to the higher reward side to follow the change in *V*^π^(*s*) (*P* < 0.01, *t* test; *n* = 8 agents; Fig. 3C). Similarly, space-tuned neurons in the mouse were more enriched in the side with higher *V*^π^(*s*) (*P* < 0.05, *t* test; *n* = 6 mice; Fig. 3C and fig. S4, A and B). Reward omission in randomly interleaved trials did not entirely eliminate neural space tuning to the reward zone (45.2 ± 11.1% remained; mean ± SEM; *n* = 5 mice), especially in those cortical regions with enriched state-value representations (Fig. 3D and fig. S4, C and D). As *V*^π^(*s*) is stable at the expert stage, we concluded that those remaining neurons not changing their spatial representations in the reward zone encoded *V*^π^(*s*) but not reward. The observed space tuning was neural substrates of *V*^π^(*s*) but not those of the state occupancy (fig. S5, A to C).

Furthermore, the open-loop experiment, when tested in the deep RL agent, led to a moderate decrease in the number of state-value neurons and almost complete removal of the policy neurons (*P* < 0.02 for the state-value and *P* < 0.001 for the policy, *t* test with Bonferroni correction; *n* = 4 agents; Fig. 3E). Notably, mouse cortical neurons showed similar degrees of reduction in the number of the state-value and policy neurons (state-value: M1: *P* = 0.18; M2: *P* = 0.18; S1: *P* = 0.79; RSC: *P* = 0.91; VISa: *P* = 0.16; VISam: *P* = 0.26; policy: M1: *P* = 0; M2: *P* = 0; S1: *P* = 0; RSC: *P* = 0; VISa: *P* = 0.001; VISam: *P* = 0 compared with 0; *n* = 9 and 7 mice for closed-loop and open-loop, bootstrap; Fig. 3E), although mice were equally engaged in the task by displaying a similar degree of joystick movement (joystick movement epoch: 38.6 ± 3.3% for closed-loop and 46.9 ± 3.6% for open-loop; mean ± SEM; *P* = 0.11, unpaired *t* test). Together, these results lend strong support that cortical neurons share fundamental representational features of the actor and critic of the deep RL agent.

### Sparse representations in the deep RL agent and mouse cortex during learning

Brain-inspired computing for machine intelligence, collectively known as neuromorphic computing, has recently garnered considerable attention to mimic the brain’s energy-efficient information processing machinery (*49*). Sparse coding has been regarded as one of the efficient mechanisms extensively studied in both neuroscience and artificial intelligence (AI) (*50*, *51*). We examined how learning affected sparseness of population activity in networks of the deep RL agent and mouse cortex.

**Fig. 4.**
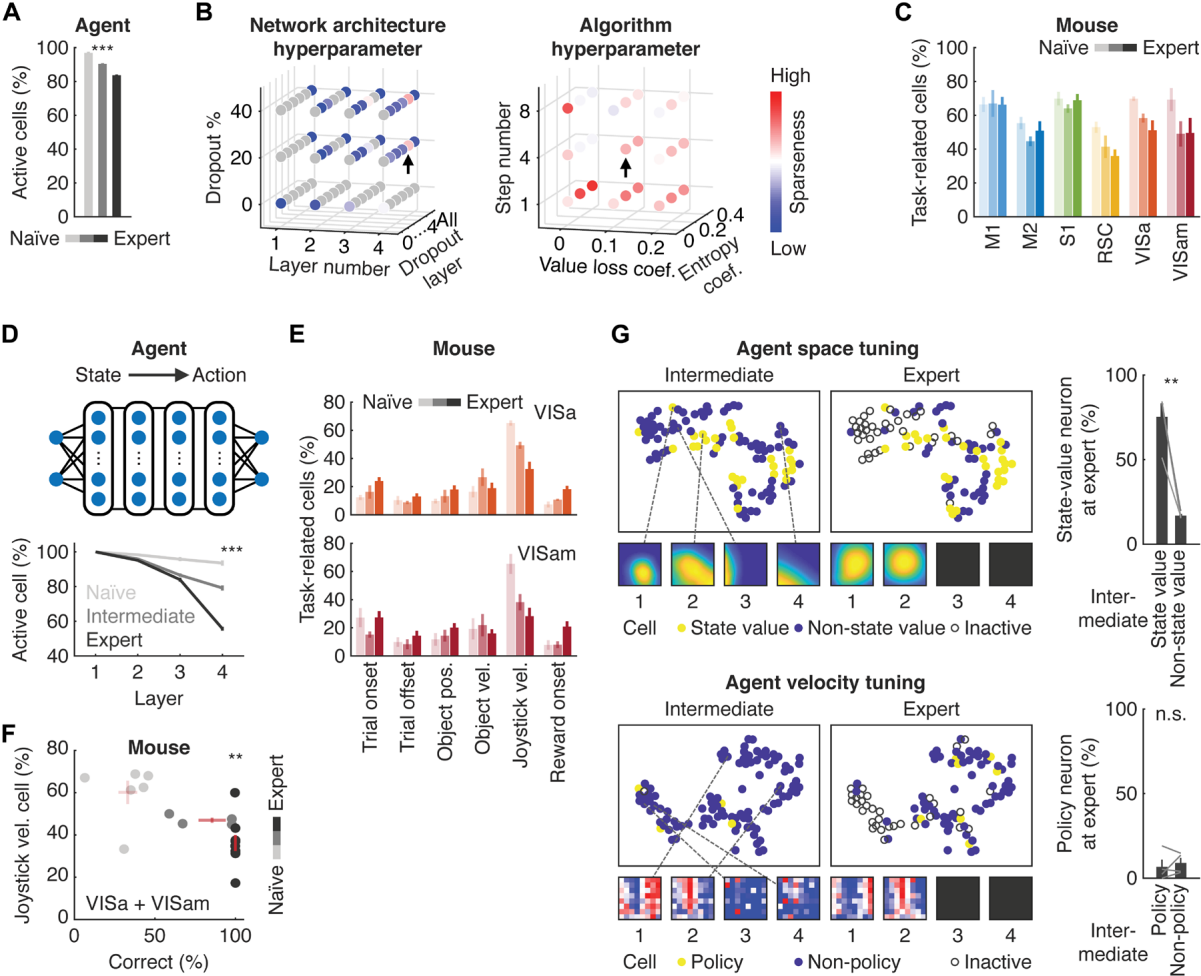
Learning-dependent sparse representations in the deep RL agent and mouse cortex. (**A**) Sparser representations in the ANN of the deep RL agent over learning as indicated by the reduction of active cells (***P < 0.001, one-way ANOVA; *n* = 4 agents). (**B**) Sparse representations in the hyperparameter space for the network architecture and algorithm. The arrows are the selected model parameters for further analysis. (**C**) Fraction of task-related neurons in different cortical regions of the mouse over learning. (**D**) Learning-dependent sparse representations in the deep RL agent are more prominent in action-related layers (***P < 0.001, two-way ANOVA for learning, layer, and interactions between the two factors; *n* = 4 agents). (**E**) Changes in fractions of neurons encoding different task variables during learning in the PPC. Note that object position and joystick velocity correspond to state and action-related task variables, respectively. (**F**) Relationship between task performance of the mice and fraction of joystick velocity-encoding neurons (*r* = −0.67, **P < 0.01, permutation test; *n* = 22 mice). (**G**) Selective elimination of non-state-value neurons and dynamic recruitment of policy neurons in the deep RL agent during learning (**P < 0.01 and n.s., *P* = 0.55, *t* test; *n* = 4 agents). Left: Each dot represents neuron’s tuning in space or velocity embedded in the respective *t*-distributed stochastic neighbor embedding (*t*-SNE) coordinate of the intermediate stage. Space and velocity tuning of example cells were shown below. Black boxes indicate inactive neurons. Right: Fraction of state-value or policy neurons at expert stage based on whether neurons were either state-value- or non-state-value-related (top) or policy- or non-policy–related (bottom) at the intermediate stage.

We first investigated neural activity in the deep RL agent over the course of learning. During learning, progressively more neurons became silent, indicating that the deep RL agent autonomously became more efficient to compute *V*^π^(*s*) and π [*P* < 0.001, one-way analysis of variance (ANOVA); *n* = 4 agents; Fig. 4A]. Hyperparameter search for the neural network architecture revealed that shared network architecture between the actor and critic and insertion of dropout in the deep layer of the network were the two key factors that augmented sparseness of the population activity (Fig. 4B and fig. S6A). The hyperparameters for the deep RL algorithm, by contrast, had less influence (Fig. 4B and fig. S6A). As individual cortical neurons showed mixed selectivity to multiple task variables, these results suggest the importance of multiplexed representations derived from the shared neural network architecture in sparse coding. Furthermore, because dropout in machine learning is considered equivalent to an unreliable response (“noise”) in biological neurons (*52*), the large influence of dropout insertion in the layer close to action nodes of the network indicates that unreliability in downstream brain regions involved in sensorimotor processing could promote sparse representations. As in the case of the deep RL agent, the fraction of task-related neurons in the mouse cortex was also reduced over learning in regions such as the RSC and PPC (M1: *P* = 0.5; M2: *P* = 0.27; S1: *P* = 0.41; RSC: *P* = 0; VISa: *P* = 0; VISam: *P* = 0.03, naïve versus expert; *n* = 6 and 9 mice for naïve and expert, bootstrap; Fig. 4C). Furthermore, deeper layers of the deep RL agent, which corresponded to downstream action-related layer, became more silent over learning (*P* < 0.001, two-way ANOVA; *n* = 4 agents; Fig. 4D). Similarly, we detected a pronounced decrease in the fraction of PPC neurons encoding joystick movement (VISa: *P* = 0.001; VISam: *P* = 0.001, naïve versus expert; *n* = 6 and 9 mice for naïve and expert, bootstrap; Fig. 4E and fig. S6B), which was correlated with the performance of the mice (*r* = −0.67, *P* < 0.01; Fig. 4F). Such sparse representations were unlikely because of mere experience with the behavior setup, as there was no correlation between the number of training sessions and the fraction of movement-related neurons in the PPC of the expert mice (*r* = −0.07, *P* = 0.85).

We further studied how neurons in the trained deep RL agent were selectively retained as active cells during learning. In the deep RL agent, the spatial coherence of the retained neurons was higher than that of inactivated neurons during learning (fig. S6C). Moreover, state-value–representing neurons at the intermediate stage remained as they were at the expert stage, while non-state-value–representing neurons at the intermediate stage tended to become inactive (Fig. 4G). Similarly, velocity-encoding neurons remained active over learning, whereas those non-velocity-encoding neurons became inactive (fig. S6C). Unlike the state-value–representing neurons, however, the identity of the policy-representing neurons dynamically changed so that a different set of velocity-tuned neurons was recruited to express the learned policy (Fig. 4G). These results demonstrate that there exists a learning-dependent mechanism to selectively retain representations of task-relevant decision variables while eliminating task-irrelevant representations in the deep RL agent, which is consistent with learning-dependent enrichment of state-value– and policy-encoding neurons in the mouse cortex.

### Representations of the subgoal in the deep RL agent and mouse cortex

One of the hallmarks of the intelligent system is its ability to decompose a task into a set of simpler subtasks with internally defined subgoals. Artificial agents have been designed to assign “intrinsic motivation” to a subgoal to solve tasks with sparse and delayed rewards with high sample efficiency (*34*, *53*). For example, in a navigation task, a bottleneck region that the RL agent passes through in successful trials is considered as a subgoal and the RL agent can autonomously discover it to enhance learning speed (*54*). We noted that, during the object manipulation task, individual mice achieved idiosyncratic solutions by exploiting a waypoint to reach the reward zone. We hypothesized that, in addition to the extrinsic water reward, mice assigned an intrinsic value to these states for efficient learning.

**Fig. 5.**
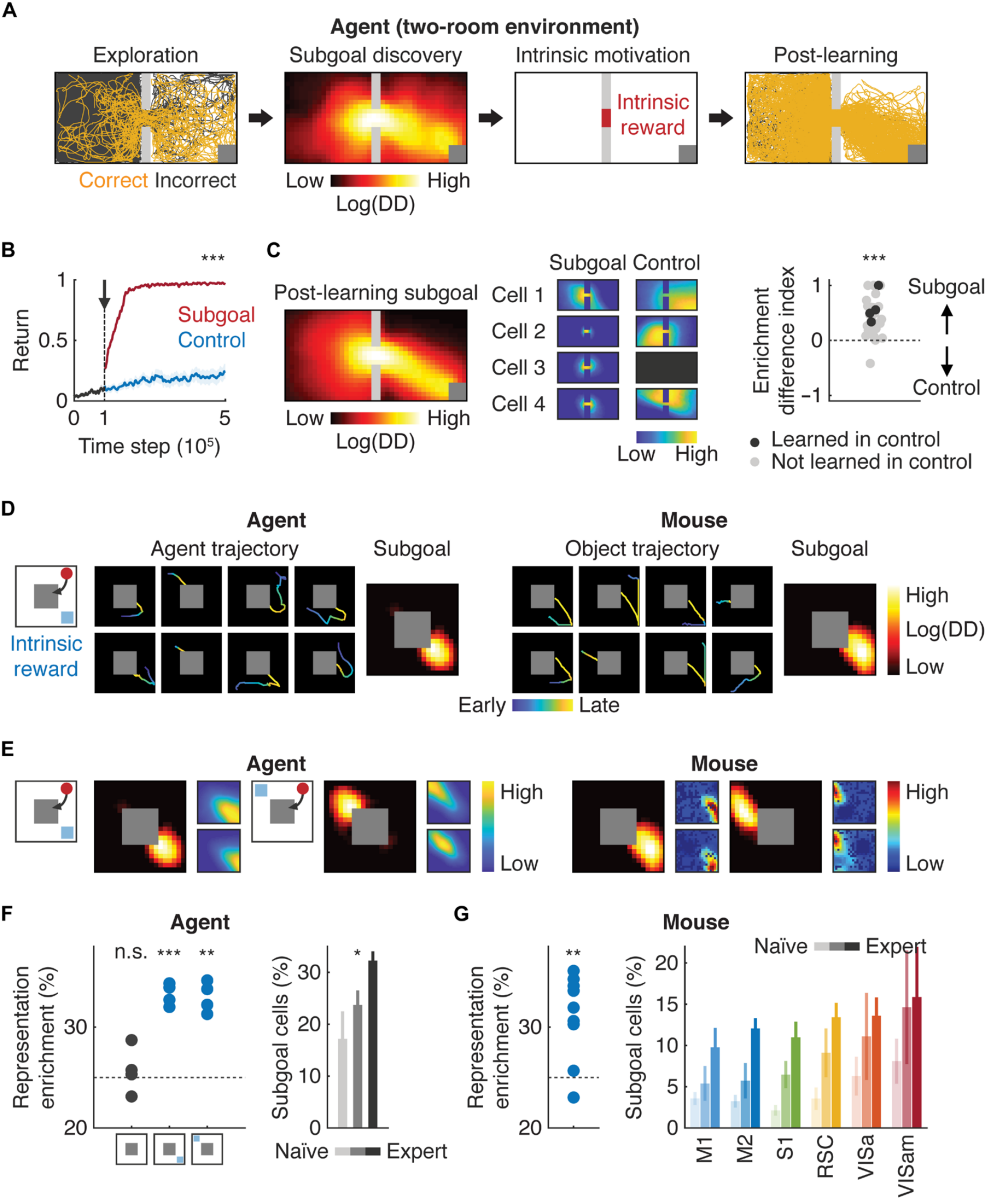
Subgoal representations in the deep RL agent and mouse cortex. (**A**) Schematic of the subgoal discovery algorithm. The agent started from random location in the left room to reach the reward zone (gray squares) in the right room. After exploration, an intrinsic reward was assigned to the subgoal identified with diverse density (DD) of trajectories. (**B**) Improved sample efficiency with the subgoal discovery algorithm (***P < 0.001, *t* test; *n* = 30 agents). The arrow indicates when the subgoal discovery or control algorithm was introduced. (**C**) Left: Example of a learned subgoal. Middle: Example space tuning of the same neurons in the deep RL agents trained with the subgoal discovery or control algorithm. The dark box indicates an inactive neuron. Right: Difference in enriched representations of the door region with the subgoal discovery versus control algorithm (***P < 0.001, *t* test compared with 0; *n* = 30 agents). (**D**) Example trajectories and subgoal of the deep RL agent with an intrinsic reward (left) and those of the mouse (right). (**E**) Example space tuning to the subgoal in the deep RL agent and mouse cortex. (**F**) Left: Enrichment of space representations in the deep RL agent in a quadrant of the arena with an intrinsic reward (n.s., *P* = 0.57; ***P < 0.001 and **P < 0.01, *t* test compared with 25% with Bonferroni correction; *n* = 4 agents). Right: Learning-dependent increase in the fraction of subgoal-encoding neurons in the deep RL agent (*P < 0.05, one-way ANOVA; *n* = 4 agents). (**G**) Left: Same as (F) in the mouse cortex in a quadrant with a subgoal (**P < 0.01, *t* test compared with 25%; *n* = 9 mice). Right: Same as (F) in different cortical regions.

To test this hypothesis, we first theoretically explored what entails subgoal representations by examining neural activity in the deep RL agent. We built a two-room environment canonically used in studies of the subgoal, where the agent was required to move from one room to the other through a door to reach the goal (Fig. 5A). After the agent explored the environment, we introduced the subgoal discovery algorithm, which uses diverse density (DD) to identify spatial bins in the arena with high density of successful trajectories and low density of unsuccessful trajectories (fig. S7A) (*54*, *55*). In the case of the two-room environment, the subgoal corresponds to a bottleneck door region based on the DD of agent trajectories. After the subgoal was identified, a value was assigned to the corresponding state to mimic intrinsic motivation. Compared to the control algorithm where the total reward size was matched, the subgoal discovery algorithm accelerated learning of the agents (*P* < 0.001, *t* test; *n* = 30 agents; Fig. 5B). We observed characteristic enrichment of neural representations in the bottleneck door region with the subgoal discovery algorithm (*P* < 0.001, *t* test; *n* = 30 agents; Fig. 5C). Agents trained with the control algorithm, though some of them eventually learned the task equally well, did not show such enriched representations (Fig. 5C and fig. S7B). These results establish that enriched spatial representations in a particular state are the signature of the subgoal where the RL agent assigned an intrinsic value.

We next trained the deep RL agent in the original task with an internal reward assigned to a region outside the reward zone to simulate intrinsic motivation (Fig. 5D). The deep RL agent with the intrinsic value behaved similarly to the object manipulated by individual mice (Fig. 5D). Neural activity in hidden layers of the ANN of the RL agent revealed spatial representations corresponding to the agent-specific subgoal, with enriched representations in a quadrant of the arena where the intrinsic value was assigned (Fig. 5, E and F). As a control, we trained RL agents without the intrinsic value, which showed uniform distribution of space tuning across the quadrants (Fig. 5F). Moreover, the fraction of subgoal-representing neurons, determined by Pearson’s correlation coefficient between the space tuning and DD, increased during learning (*P* < 0.05, one-way ANOVA; *n* = 4 agents; Fig. 5F).

The results obtained with the deep RL agent led us to reason that if the mouse cortex represents a subgoal, an internal value assigned to the subgoal state can be revealed by examining its enriched spatial representations. Trajectory analysis of the object revealed emergence of animal-specific subgoals during learning. Because of the inherent bias in the object movement direction (top left and bottom right direction, fig. S1A), mice gradually learned to move the object to the bottom right or top left corner of the arena before reaching the reward zone (Fig. 5D and fig. S7C). Neural representations of space were enriched in a quadrant of the arena with a corresponding subgoal (Fig. 5, E and G). We found that 39.3% of space-tuned neurons (*n* = 5273 cells) were statistically (*P* < 0.05) corresponding to subgoal representations (expected percentage by chance assuming uniform distribution of space tuning: 28.8%; *P* < 0.001), and subgoal-representing neurons became more enriched during learning (M1: *P* = 0.001; M2: *P* = 0; S1: *P* = 0.001; RSC: *P* = 0; VISa: *P* = 0.01; VISam: *P* = 0.09, naïve versus expert; *n* = 6 and 9 mice for naïve and expert, bootstrap). These neurons were modestly more enriched in the PPC relatively to other regions (VISa: *P* = 0.03, 0.21, 0.12, and 0.42; VISam: *P* = 0.06, 0.25, 0.16, and 0.28 compared with M1, M2, S1, and RSC, respectively; *n* = 9 mice, bootstrap; Fig. 5G). These results demonstrate that mice gradually learned to exploit a waypoint as a subgoal by assigning an intrinsic value, potentially to accelerate learning. Together, our observation indicates that cortical neurons represent the value of each state, regardless of whether it is extrinsic or intrinsic.

### Functional convergence between neural networks of the deep RL agent and PPC for sensorimotor integration

**Fig. 6.**
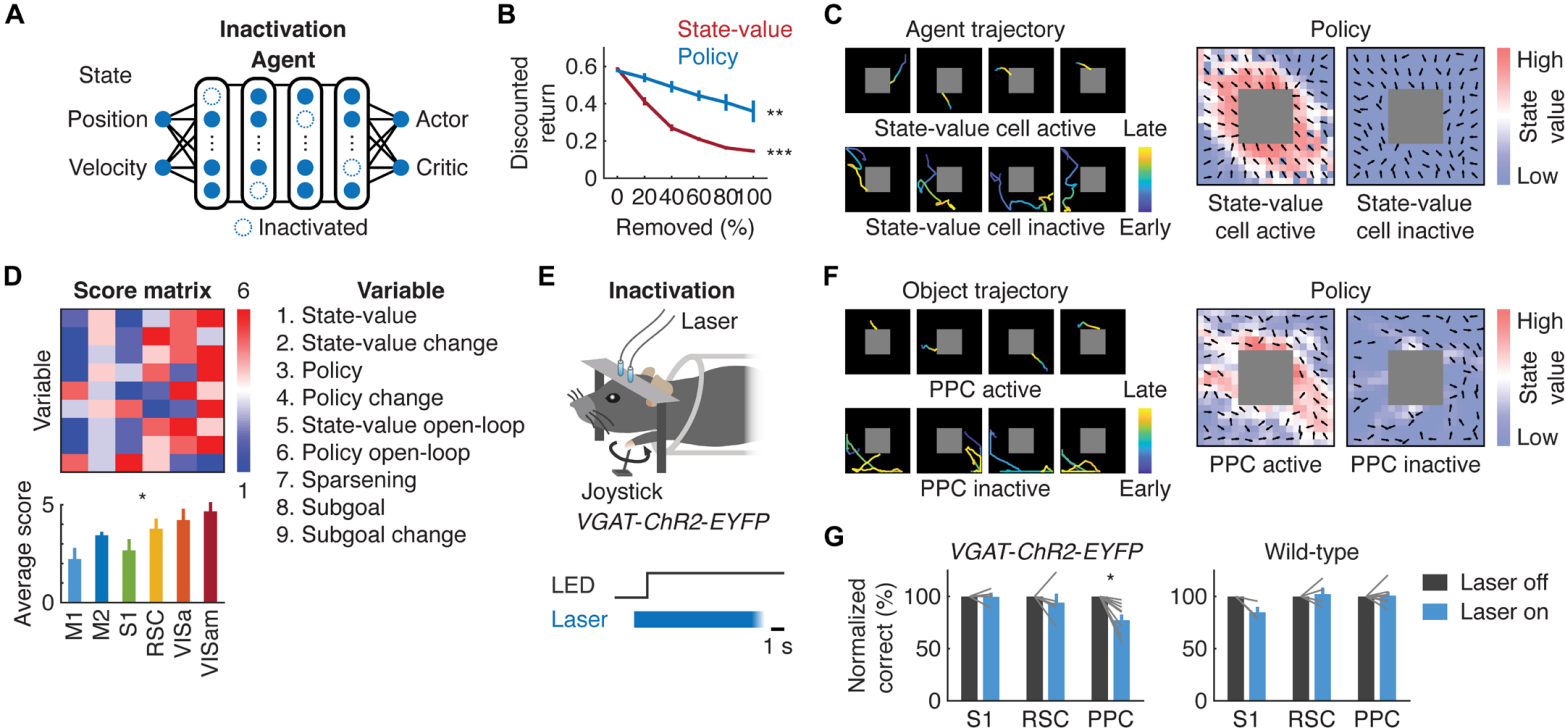
Functional convergence between the ANN of the deep RL agent and mouse PPC. (**A**) Schematic of inactivation of state-value or policy neurons in the deep RL agent. (**B**) Decrease in the task performance as a function of inactivated fractions of state-value or policy neurons (***P < 0.001 and **P < 0.01, one-way ANOVA; *n* = 4 agents). (**C**) Example trajectories (left) and state-value function and policy (right) of the deep RL agent when the state-value neurons were intact or inactivated. (**D**) Score for each variable measured throughout the study in each cortical region. Scores are based on ranks derived from neural correlates of these variables in the deep RL agent (*P < 0.05, Kruskal-Wallis test; *n* = 9 variables). (**E**) Schematic of optogenetic suppression of neural activity. In 20% of randomly interleaved trials, the blue laser was turned on for 10 s starting 1 s before the trial onset. (**F**) Same as (C) of the object when the PPC neurons were intact or inactivated. (**G**) Task performance in VGAT-ChR2-EFYP (left) or wild-type (right) mice with the laser on or off (VGAT-ChR2-EYFP: *P* > 0.05, *n* = 4 mice for S1; *P* > 0.05, *n* = 5 mice for RSC; *P < 0.05, *n* = 9 mice for PPC, two-way ANOVA with post hoc comparisons; *P* < 0.05 for interaction between cortical regions and optogenetic manipulations; wild-type: *P* > 0.05, *n* = 3 mice for S1; *P* > 0.05, *n* = 4 mice for RSC; *P* > 0.05, *n* = 7 mice for PPC, two-way ANOVA with post hoc comparisons; *P* > 0.05 for interaction between cortical regions and optogenetic manipulations).

Last, we tested whether the enriched neural representations of the state-value and policy were critical for the task performance. In the deep RL agent, we silenced the activity of state-value– and policy-encoding neurons and tested its consequence on the agent’s performance (Fig. 6A). The task performance of the agent deteriorated over the increasing number of silenced state-value– or policy-representing neurons (state-value–representing cells: *P* < 0.001; policy-representing cells: *P* < 0.01, one-way ANOVA; *n* = 4 agents) with accompanying effects on its trajectories, *V*^π^(*s*) and π (Fig. 6, B and C).

The enriched neural correlates of the RL variables in the PPC point to its important role in the actor-critic algorithms (Fig. 6D). We therefore suppressed the activity of neurons in the PPC of transgenic mice (VGAT-ChR2-EYFP) with optogenetic stimulation of Channelrhodopsin-2 (ChR2)–expressing inhibitory interneurons, which led to a significant decrease in the task performance (normalized correct rate relative to laser-off trials: 99.7 ± 3.9%, *n* = 4 mice for S1; 94.1 ± 8.5%, *n* = 5 mice for RSC; 77.3 ± 5.5%, *n* = 9 mice for PPC; mean ± SEM; *P* < 0.05 for PPC and interaction between cortical regions and optogenetic manipulations, two-way ANOVA; Fig. 6, E to G). The task performance was not affected in wild-type control mice receiving the same laser pulses [normalized correct rate relative to laser-off trials: 84.7 ± 5.4%, *n* = 3 mice for S1; 102.3 ± 6.3%, *n* = 4 mice for RSC; 100.7 ± 3.9%, *n* = 7 mice for PPC; mean ± SEM; not significant (n.s.) for all regions and interaction, two-way ANOVA; Fig. 6G]. Inactivation of the PPC also led to similar decline in the object trajectories, *V*^π^(*s*) and π (Fig. 6F). As the deep RL agent entails an “end-to-end” framework relating sensory information with appropriate actions, these results suggest that there exist close correspondences between neural networks of the deep RL agent and PPC in representation learning underlying sensorimotor integration.

## DISCUSSION

AI research attempts to design intelligent machines that can think and act like humans (*36*). To this end, numerous RL algorithms have been developed to imitate high sample efficiency of humans and other animals. Whether the brain shares similar algorithmic features has been an open question. Understanding the brain may, in turn, provide new opportunities to build more intelligent machines. In this study, we designed a behavior paradigm with high-dimensional state and action space for mice and directly compared neural representations in the artificial and biological neural networks. Representations in the mouse PPC shared marked similarities with those in the ANN of the RL agent for value estimation and policy computation when the RL agent was optimized for its task performance. Given the proposed role of the PPC in sensorimotor integration (*27*) and the “end-to-end” neural architecture of the deep RL agent (*28*, *29*), these results uncovered the functional convergence between the two systems in learning-related representation of actor-critic algorithms.

Learning in the artificial and biological systems both led to sparse representations. Such sparse representations were facilitated when the critic and actor networks were shared. As the individual cortical neurons showed mixed selectivity to encode multiple task variables, this result indicates a functional role of multiplexed representations in sparse coding. Furthermore, although it is generally accepted that dropout induces sparse representations, introduction of dropout in the layer close to action nodes of ANN in the deep RL agent further promoted sparse coding, suggesting that the noise in the brain could contribute to energy-efficient information processing machinery. We propose that such hyperparameter tuning of the deep RL agent to imitate neural representations in the brain is a useful approach to design more efficient systems.

Our analysis of cortical representations revealed that some of the sample efficient RL algorithms were potentially realized in the mouse cortex, especially in the PPC. Subgoal representations, for example, are considered useful to solve problems with sparse and delayed rewards. Learned subpolicies optimized for each subgoal can be hierarchically recomposed to solve novel tasks (*53*). Human studies demonstrated that the brain encodes subgoal-related error signals (*16*). Here, we provide, to the best of our knowledge, the first evidence for learning-dependent emergence of neural representations of the subgoal in single neurons. Note that the observed subgoal representations may be linked to other decision variables than intrinsic motivation. With additional task designs to measure and manipulate intrinsic values in mice, future studies will address the significance of these representations.

Notably, although we focused on decision variables related to reward-based learning, other types of learning, such as those driven by sensory prediction errors (*56*), could also account for some of the observed representations in the mouse cortex. In other words, representation similarities between the artificially and biologically intelligent systems by no means prove that the brain uses the same algorithm. Nonetheless, the dependence of these representations on the reward function suggests that they were at least partially driven by reward-based learning. Unentangling precisely which representations derive from reward-based learning or sensory prediction error–driven learning awaits further investigation. Together, our approach to validate RL algorithms using the existing proof of intelligence of the brain supports their plausibility as a candidate mechanism to better recapitulate animal-like cognitive flexibility in machines.

## MATERIALS AND METHODS

### Animals

All procedures were in accordance with the Institutional Animal Care and Use Committee at Nanyang Technological University (protocol number: A0352). Wild-type (C57BL/6J) and transgenic mice were obtained from Charles River Laboratories and the Jackson Laboratory (CaMKII-tTA: 007004; TRE-GCaMP6s: 024742; VGAT-ChR2-EYFP: 014548), respectively. Mice were housed in a reversed light cycle (12 hours:12 hours) in standard cages, and the experiments were typically performed during the dark period. Both male and female hemizygous mice were used. Sample size was determined on the basis of the standard in the field. Mice were randomly allocated to each group, and no blinding was performed.

### Surgery

Adult mice (between 7 weeks and 4 months old) were anesthetized with 1 to 2% isoflurane and a circular piece of scalp was removed. For surgery of the imaging experiment, after the underlying bone was cleaned with a razor blade, a craniotomy (~7 mm in diameter) was made around the bregma with a dental drill and an imaging window was placed in the craniotomy. The imaging window was constructed from a small (~6 mm in diameter) glass plug (#2 thickness; Fisher Scientific, 12-540-B) attached to a larger (~8 mm in diameter) glass base (#1 thickness; Fisher Scientific, 12-545-D) using an ultraviolet curing adhesive (Norland, NOA 61). Agarose (1.5%) (Sigma-Aldrich, A9793-50G) was applied to fill the gap between the skull and window. A custom-built titanium headplate was then implanted on the window with cyanoacrylate glue and cemented with the black dental acrylic (Lang Dental, 1520BLK or 1530BLK). Buprenorphine (0.05 to 0.1 mg/kg of body weight), Baytril (10 mg/kg of body weight), and dexamethasone (2 mg/kg of body weight) were subcutaneously injected, and mice were monitored until they recovered from anesthesia. Mice were excluded when the optical window was occluded.

For surgery of the optogenetics experiment, optic fiber sleeves (Thorlabs, ADAL1) were bilaterally attached over the skull of VGAT-ChR2-EYFP or wild-type mice with cyanoacrylate glue with the following coordinates: S1 (2.0 mm posterior and 3.8 mm lateral to the bregma), RSC (2.0 mm posterior and 0.4 mm lateral to the bregma), and VISa (2.0 mm posterior and 1.8 mm lateral to the bregma). The black dental acrylic (Lang Dental, 1520BLK or 1530BLK) was then applied, and buprenorphine (0.05 to 0.1 mg/kg of body weight) and Baytril (10 mg/kg of body weight) were subcutaneously injected.

### Behavior tasks

At least 3 days after the surgery, mice were water-restricted for ~2 weeks. They were then trained to perform the object manipulation task using a custom-made joystick. The internal springs of the joystick (APEM, M11L061P) were changed to the ones with a spring rate of 0.048 N/mm (RS Pro, 821-396). A brass rod (K&S Precision Metals, 8162) with Parafilm (Fisher Scientific, FIS#13-374-16) was attached as a handle so that the mouse could manipulate the joystick easily with its right forepaw. Another brass rod was also placed for the left forepaw to rest on. The object in the arena was a 525-nm LED (Thorlabs, LED525E) attached to a three-dimensional printed cube. The object was moved on a custom-made aluminum support attached to a pair of slide potentiometers (Bourns, PSM01-081A-103B2) and driven by the joystick controlled with Arduino Leonardo (Arduino) and a motor shield (Adafruit, 1438). The arena (10 cm × 10 cm) was made of MakerBeam (SGBotic, HDW-02309), and the center of the reward zone was indicated by another 525-nm LED (Thorlabs, LED525E). Licking events were detected by a custom-made touch sensor. The task structure was controlled by Bpod (Sanworks) using custom codes written in MATLAB, and all the task variables were measured by WaveSurfer (Janelia Research Campus) at the sampling rate of 2000 Hz.

The training protocol started with a few days of habituation to head restriction, which was followed by four training phases. At the first phase, mice received a water (8 μl) reward when they moved the object in any direction using the joystick. At the second phase, the reward zone was set to be 8 cm × 8 cm in the middle of the arena, and mice were trained to move the object toward the reward zone to receive a reward. At the third and fourth phases, the size of the reward zone was further reduced to 6 cm × 6 cm and to 4 cm × 4 cm, respectively. Mice performed up to 60 trials over ~1 hour for each session, and when they completed two consecutive sessions successfully (all 60 trials within 30 min), they moved to the following phase. In unsuccessful sessions, training was manually intervened. The intermediate learning stage was defined as the time when the fourth training phase (reward zone of 4 cm × 4 cm) was first introduced, while the expert learning stage was defined as the time when mice successfully completed at least two consecutive sessions of the fourth phase. At the naïve learning stage, the environment with the reward zone of 4 cm × 4 cm was used without going through the first three phases of the training. The whole training procedure took approximately 2 to 3 months for each animal.

For all the training stages, the trial started with the onset of the object and target LEDs. When the object reached the reward zone, the LEDs were turned off, which was followed by 4 s of a reward consumption period and 2 s of an intertrial interval (ITI). The object was made stationary once the reward zone was reached. After each successful trial, the object was reinitialized to a random position outside the reward zone. Each trial lasted up to 5 min, and when the object failed to reach the reward zone, an ITI immediately followed.

The experiment with two-reward magnitudes followed the same task structure with the fourth phase of the training (reward zone of 4 cm × 4 cm), except that the reward size was made different depending on which side of the reward zone the object reached. For example, when the object reached one side of the reward zone (right or bottom side depending on mice), mice received 10 μl of water, whereas if it reached the other side (left or top side depending on mice), they received only 1 μl of water. Four mice were used in the environment where the reward was high on the right side of the reward zone, and two mice were used in the environment where the reward was high on the bottom side of the reward zone. The experiment with an interleaved reward also followed the same task structure, except that there were 120 trials per session, and the reward was randomly omitted in half of the trials within each session, although mice successfully moved the object to the reward zone. The open-loop experiment (decoupling between the joystick and object movement) also followed the same task structure, except that the object was moved randomly in one of eight directions at various speeds every 100 ms regardless of the joystick movement. A reward was provided when the object reached the reward zone. These three experiments were conducted on expert mice.

### Two-photon calcium imaging

Images were acquired using 2p-RAM (Thorlabs) controlled with ScanImage (Vidrio Technologies) with a laser (InSight X3, Spectra-Physics) whose excitation wavelength was tuned to be 940 nm with the power of ~40 mW at the objective lens. The frame rate was ~5.67 Hz, and the imaging resolution was 1 × 0.4 pixel/μm with two fields of view of 0.5 mm × 5 mm at the depth of ~200 to 300 μm (layer 2/3). Imaging frames were recorded with WaveSurfer and aligned to behavior events offline. The same cortical regions were imaged three times, and results were combined for the expert mice. Fields of view were then moved to other regions as long as the imaging areas were not occluded. Between 3 and 12 sessions were imaged for expert animals (3 sessions for three mice, 9 sessions for two mice, and 12 sessions for four mice), 3 sessions for intermediate animals (seven mice), between 2 and 8 sessions for naïve animals (2 sessions for one mouse, 6 sessions for one mouse, and 8 sessions for four mice), between 6 and 8 sessions for the two-reward-magnitude experiment (6 sessions for three mice and 8 sessions for three mice), between 4 and 8 sessions for the interleaved-reward experiment (4 sessions for one mouse, 5 sessions for one mouse, 6 sessions for two mice, and 8 sessions for one mouse), and between 1 and 4 sessions for the open-loop experiment (1 session for one mouse, 3 sessions for two mice, and 4 sessions for four mice).

### Optogenetics

At the expert stage of the behavior training for VGAT-ChR2-EYFP mice, 20% of randomly chosen trials were assigned as optogenetics trials, and the remaining 80% of the trials were used as control trials. A 9-s laser square pulse with 1-s taper at the wavelength of 473 nm (Shanghai Laser and Optics Century, BL473T8-100FC) and at the power of ~2.9 mW/mm^2^ (Shanghai Laser and Optics Century, BL473T8-100FC) was delivered to the cortex through the sleeve attached to the skull 1 s before the optogenetics trial onset. ITIs for the optogenetics experiment were extended to 6 s to ensure that the laser pulse was turned off before the next trial. To make sure that the effects were not due to other variables, such as heat or light, wild-type mice were also trained for ~2 to 3 months and the same procedure was followed as the VGAT-ChR2-EYFP mice.

### Behavior analysis of the mouse

#### Object trajectory and joystick movement

Object trajectories were displayed every 10 ms, and the object’s traveled distance was computed by sampling every 100 ms. Histogram of the object movement angle was obtained with 10° bins.

The angle of the initial joystick movement at each trial onset was determined by obtaining joystick deflection over 100 ms and sorted by the initial object location (top left, top right, bottom right, and bottom left). For each quadrant, the angle values over trials were sorted to 30° bins and histogram was obtained. For each mouse, general movement bias was determined as the mean angle frequency regardless of the initial position, and it was subtracted from the histogram.

Shuffled object trajectories were determined by sampling random joystick movements and obtaining corresponding object movements. Object movements that reached the edges were not considered to avoid clipping effects. Once object movements were sampled, corresponding vectors were cumulatively summed from the initial object location for each trial. If the object moved beyond the edge, it was relocated to the edge. To determine whether mice moved the joystick using visual feedback or randomly to reach the reward zone, the total object distance traveled divided by the distance between the initial object location and the reward zone was used as a metric to compare between actual and shuffled trajectories.

#### State-value function

The state-value function *V*^π^(*s_t_*) was defined as the mean discounted time steps for each spatial bin and calculated by

$$V^{\pi }(s_{t})=E[R_{t}+\gamma R_{t+1}+\gamma^{2}R_{t+2}+\cdots +\gamma^{T-t}R_{T}]$$ (6)

where E is expectation, *R_t_* is a reward at the time point *t* taken every 10 ms, γ is a discount factor set to be 0.99, and *T* is a trial end when the mouse receives a reward of 1. Because the animal did not receive a reward elsewhere other than the reward zone, the state-value function can be simplified as (*38*)

$$V^{\pi }(s_{t})=E[\gamma^{T-t}]$$ (7)

The state-value of the reward zone was set to be 1.

For the two-reward-magnitude experiment, *V*^π^(*s_t_*) was calculated in the same way, except that the reward size was altered according to the amount of water given to the mouse relative to the original task (1.25 and 0.125 for the high and low reward side of the reward zone, respectively).

#### Policy

Policy π was defined as the probability distribution of object movement direction. π was plotted as the preferred action *a* (i.e., preferred object movement) in a given state *s* and calculated by the vectorial sum of all velocity vectors for each spatial bin. Each velocity vector was determined on the basis of the angle and speed of the object movement over 100 ms.

#### Subgoal

A subgoal was determined by computing log(DD) of successful and unsuccessful trials according to the previous study (*54*). The most diversely dense region of the arena is the spatial bin *x* where the object passed with multiple successful trajectories and not with unsuccessful ones. The distance *d*_*i*,*j*_ between each spatial bin *x* of the arena (20 × 20 bins) and each point *j* of a 10-ms binned trajectory in each trial *i* was used as approximation to the relative probability *p*_*i*,*j*_ that, given a spatial bin *x*, corresponds to a subgoal

$$p_{i,j}=e^{\left(-{\left\|d_{i,j}\right\|}^{2}\right)}$$ (8)

*p*_*i*,*j*_ was bounded between 0 and 1 and higher when the distance between *x* and *j* was shorter. For each *x*, the joint probability was computed across different points on the trajectory for successful and unsuccessful trials according to

$$p(x|T_{i}^{+})=1-\prod_{1\leq j\leq q}(1-p_{i,j}^{+})\;(\text{successful trials})$$ (9)

$$p(x|T_{i}^{-})=\prod_{1\leq j\leq q}(1-p_{i,j}^{-})\;(\text{unsuccessful trials})$$ (10)

where *q* is the number of 10-ms binned points on the trajectory. DD was calculated according to

$$\text{DD}(x)=\prod_{1\leq i\leq m}p(x|T_{i}^{+})\prod_{1\leq i\leq n}p(x|T_{i}^{-})$$ (11)

where *m* and *n* are the numbers of successful and unsuccessful trials, respectively.

To study how the mouse developed subgoal representations, log(DD) was determined in each session of the last behavior training phase (4 cm × 4 cm reward zone) and compared to log(DD) of the last session using Pearson’s correlation coefficient according to

$$r=\frac{\sum (x_{i}-\bar{x})(y_{i}-\bar{y})}{\sqrt{\sum {\left(x_{i}-\bar{x}\right)}^{2}\sum {\left(y_{i}-\bar{y}\right)}^{2}}}$$ (12)

where *x_i_* and *y_i_* are log(DD) in the spatial bin *i* in two different sessions. $\bar{x}$ and $\bar{y}$ are mean values of the respective variables. The correlation coefficients of the first, middle, and last five sessions (excluding the last session) were averaged and categorized as early, intermediate, and late.

#### Optogenetics

Behavior for the optogenetics experiment was analyzed during the 9 s from the trial onset when the laser was on in the optogenetics trials or during the equivalent period for the control trials. After the laser was turned off in each optogenetics trial, mice typically completed the trial within 5 min. Sessions in which all 60 trials were not completed within 45 min were excluded from the analysis.

### Imaging data processing

To obtain deconvolved calcium traces from cell bodies, we applied Suite2p (https://github.com/cortex-lab/Suite2P) to perform image registration, semiautomated cell detection, and neuropil correction (*45*). Only those neurons whose activity passed a threshold of 20 at least once were further analyzed. To test consistency of the results, we applied other deconvolution methods such as MLspike (*47*) and LZero (*46*). For MLspike, neuropil-subtracted calcium signals were fitted to the algorithm using its automatic baseline detection and recommended parameters for GCaMP6s. For LZero, *df*/*f* computed with Suite2p was fitted to the algorithm. The parameters γ = 0.9 and λ = 0.1 were determined by grid search using the mean squared error between raw and reconstructed calcium traces as a metric to evaluate the fitting. The deconvolved spikes were summed over three frames to match the nonbinary signals used in other methods.

Parcellation of the cortical areas was based on the Allen Mouse Common Coordinate Framework. Each neuron was categorized to one of six cortical regions based on the distance from the bregma, which was located at the center of the imaging window. Neurons that were located at the border of the cortical areas were not categorized (expert: M1: 7467 neurons; M2: 6259 neurons; S1: 7562 neurons; RSC: 8664 neurons; VISa: 660 neurons; VISam: 632 neurons; intermediate: M1: 3081 neurons; M2: 3336 neurons; S1: 2900 neurons; RSC: 2997 neurons; VISa: 994 neurons; VISam: 290 neurons; naïve: M1: 2325 neurons; M2: 3770 neurons; S1: 1763 neurons; RSC: 2602 neurons; VISa: 364 neurons; VISam: 116 neurons; two-reward magnitude: M1: 4037 neurons; M2: 19,979 neurons; S1: 1937 neurons; RSC: 13,070 neurons; VISa: 2551 neurons; VISam: 3447 neurons; interleaved reward: M1: 2637 neurons; M2: 4040 neurons; S1: 1252 neurons; RSC: 3043 neurons; VISa: 162 neurons; VISam: 70 neurons; open-loop: M1: 1508 neurons; M2: 1060 neurons; S1: 1974 neurons; RSC: 8147 neurons; VISa: 1168 neurons; VISam: 411 neurons).

#### Generalized linear model

Neural encoding of experimentally designed task variables was modeled with the GLM for each neuron independently (*40*, *41*). We used Poisson GLM to compute weights of all measured task variables in predicting activity of single neurons based on the deconvolved calcium signal (*57*). The task variables included the trial onset and offset times, object velocity, object position, joystick velocity, and reward onset times. Because the task variables were measured at a higher temporal sampling rate (2000 Hz) than the imaging (5.67 Hz), they were down-sampled by averaging during each imaging frame to match the imaging sampling rate.

The design matrix for the GLM was obtained as follows. The trial onset times, trial offset times, and reward onset times were represented as boxcar functions, where a value of one was assigned to these times and zero elsewhere. The angle of the object velocity and joystick velocity was discretized to eight equally spaced bins (0°, 45°, 90°, 135°, 180°, 225°, 270°, and 315°) and generated eight time series data with amplitude of the movement. The object position was calculated by binning the arena into 10 ×10 spatial bins. Each of the task variables was convolved with a set of behaviorally appropriate spatial or temporal basis functions to produce task predictors. For the trial onset and offset times, we used six evenly spaced raised cosine functions extended 2 s forward and backward in time. For object and joystick velocity, we used six evenly spaced raised cosine functions for each directional bin extended 2 s forward and backward in time. For the object position, we used 100 (10 × 10) evenly spaced raised cosine functions along the two axes of the arena. For the reward onset time, we used nine evenly spaced raised cosine functions extended 4 s forward and 2 s backward in time. The total of 217 task predictors was used to predict the deconvolved calcium signal for each neuron.

#### GLM fitting

All task predictors were *z*-scored before fitting the GLMs. The data were divided into training dataset (70% of image frames) and test dataset (30% of image frames). To avoid overfitting, GLMs were fitted to each neuron’s activity using the lassoglm function in MATLAB with fivefold cross-validation of the training data with elastic net regularization, which uses both lasso and ridge regularization with a ratio of α set to be 0.9 (0.9 lasso regularization and 0.1 ridge regularization). Lasso regularization allows the model to select a relatively small number of task predictors out of many potentially correlated predictors, whereas ridge regression distributes coefficients to correlated predictors. Together, elastic net regularization with some high α removes any degeneracies caused by strong correlations (*58*). The number of λ in the lassoglm function was set to be 100. GLM model performance was assessed for the test dataset by quantifying the pseudo-E.V. of the model according to

$$\text{Pseudo-E.V.}=1-\frac{D(\hat{y})}{D(\bar{y})}$$ (13)

where

$$D(\hat{y})=\text{log}L(y)-\text{log}L(\hat{y})$$ (14)

is a deviance from the saturated model in terms of log likelihoods, whereas

$$D(\bar{y})=\text{log}L(y)-\text{log}L(\bar{y})$$ (15)

is a deviance from the null model (*42*). The null model was calculated on the basis of the mean activity. We defined task-related cells as neurons whose pseudo-E.V. was positive and statistically higher (*P* < 0.001) than that obtained by shuffling the task predictors 1000 times with 2-s bins.

For the interleaved reward experiment, the session was split into rewarded and nonrewarded trials. GLMs were fitted independently.

#### GLM-derived response profiles for each task variable

The GLM estimates neural activity by exponentiation of weighted sum of the task predictors. Therefore, the estimated neural activity can be expressed as multiplication of the exponentiated task variables (i.e., *e*^*a* + *b*^ = *e^a^* × *e^b^*) and decomposed into activity contributions of each task variable (*44*). The model-derived response profile for a given variable can then be defined as a tuning curve for that variable by marginalizing out the effect of the other variables. We defined a task variable to be a contributor for the activity of task-related cells when removal of the variable reduced the overall pseudo-E.V., and this reduction was statistically significant (*P* < 0.05 with the Benjamini-Hochberg false discovery rate) by shuffling the task predictors 1000 times with 2-s bins.

#### Analysis of the state-value function, state occupancy, and subgoal representation in the mouse cortex

To test whether the spatial tuning of each neuron corresponded to the state-value function, state occupancy, or subgoal representations, we calculated Pearson’s correlation coefficient between the space tuning and state-value function, state occupancy (state-visit frequency in 20 × 20 bin of the arena), or subgoal [log(DD)]. We shuffled the spatial bins of object trajectories 1000 times and computed shuffled space tuning for each neuron. *P* values were computed with Pearson’s correlation coefficient between the shuffled space tuning and state-value function, state occupancy, or subgoal. We considered a neuron deemed to be representing the state-value function, state occupancy, or subgoal when the *P* value was less than 0.05 with the Benjamini-Hochberg false discovery rate.

We determined whether the fraction of neurons representing the state-value function or subgoal was above the chance level, which was based on uniform distribution of spatial tunings across the arena. We obtained Pearson’s correlation coefficient of randomly chosen spatial basis functions used for GLM with the actual state-value function or subgoal. The spatial basis functions were proxies for place fields and tiled the entire arena. We shuffled the spatial bins of the spatial basis function 1000 times and obtained *P* values for each spatial basis function. The fraction of spatial basis functions correlated with the state-value function, or subgoal was then determined using the same criteria as above. This procedure was repeated 1000 times to calculate a *P* value of obtaining the actual fraction.

For the two-reward-magnitude experiment, it was determined whether the peak location of space tuning was on the high reward (right or bottom depending on mice) side or low reward (left or top depending on mice) side of the arena. The enrichment index was then calculated as

$${\text{Enrinchment index}}_{\text{two}-\text{reward}}=\frac{{\text{Fraction of neurons}}_{\text{right or bottom side}}-{\text{Fraction of neurons}}_{\text{left or top side}}}{{\text{Fraction of neurons}}_{\text{right or bottom side}}+{\text{Fraction of neurons}}_{\text{left or top side}}}$$ (16)

As a control, the enrichment index was also determined across control sides. For example, if the right side of the reward zone was assigned as a high reward side, the enrichment index was determined by comparing the bottom side with the top side. Similarly, if the bottom side of the reward zone was assigned as a high reward side, the enrichment index was determined by comparing the right side with the left side. As the object more frequently reached the right (high reward) side of the reward zone from the bottom side of the arena or the bottom (high reward) side of the reward zone from the right side of the arena, the enrichment index for the control was slightly positive.

To determine whether the distribution of space tuning was skewed toward a quadrant with the highest log(DD) corresponding to a subgoal, the peak location of space tuning was determined for each space-tuned neuron. The representation enrichment was measured as the fraction of neurons residing in the quadrant of interest among summed fractions over all the quadrants.

For the open-loop experiment, the fraction of state-value–representing neurons was determined for closed-loop and open-loop environments, and the normalized change was calculated as

$$\text{Normalized change}=\frac{{\text{Fraction of neurons}}_{\text{open}-\text{loop}}-{\text{Fraction of neurons}}_{\text{closed}-\text{loop}}}{{\text{Fraction of neurons}}_{\text{open}-\text{loop}}+{\text{Fraction of neurons}}_{\text{closed}-\text{loop}}}$$ (17)

#### Analysis of the policy representation in the mouse cortex

To study policy representations, only conjunctive neurons encoding the space and velocity of the controlled object were analyzed. A neuron was deemed to be representing the policy when the direction tuning of the neuron was similar to object’s action distribution over eight directions in spatial bins corresponding to the top 5% of activity. The object’s action distribution was obtained by averaging movement distributions over spatial bins where movement distribution for each spatial bin was weighted by the normalized activity level in the same bin. Pearson’s correlation coefficient was calculated between the direction tuning of each neuron and the resulting direction distribution of the object. *P* values were obtained by shuffling the object movement direction 1000 times, computing shuffled direction tuning, and calculating Pearson’s correlation coefficient between the shuffled direction tuning of each neuron and the object’s direction distribution. Neurons with a *P* value of less than 0.05 with the Benjamini-Hochberg false discovery rate were considered to be policy-representing cells.

To determine whether the fraction of neurons representing the policy was above the chance level, we paired the spatial basis function used for GLM (uniform distribution across the arena) and the direction tuning of each conjunctive neuron given the same object trajectories. For each pair, the same calculation was performed as above to compute Pearson’s correlation coefficient between the direction tuning of each neuron and the resulting direction distribution of the object. This procedure was repeated 1000 times to calculate a *P* value of obtaining the actual fraction of policy-representing neurons. For the open-loop experiment, the fraction of policy-representing neurons was determined for closed-loop and open-loop environments and the normalized change was calculated according to Eq. 17.

#### Scoring cortical regions

To determine which cortical regions were similar to the ANN of the task-performance-optimized deep RL agent, neural representations of the examined variables were compared across the six cortical regions. A score of 1 to 6 (the worst to the best, respectively, based on the rank) was assigned to each cortical region for a given variable. For the state-value, policy, and subgoal, each region was ranked on the basis of their values. For the learning-dependent changes in the state-value, policy, active (sparse code), and subgoal cell fractions, Pearson’s correlation coefficient was calculated between changes in their neural representations in the mouse and those in the deep RL agent, and each region was ranked on the basis of the correlation coefficients. For the open-loop experiment, changes in the state-value and policy cells were considered, and each region was ranked on the basis of their values. The mean score was then calculated over variables for each region.

### Deep RL

#### Environment

A custom OpenAI’s gym environment was created with continuous state and discrete action space to simulate the object manipulation task for mice. The states were the agent’s position in *x* and *y* coordinate and velocity. To recapitulate the biased movement of the object in the deep RL agent (fig. S1A), we used 65 actions (none and combinations of eight speed and eight direction bins: 0°, 45°, 90°, 135°, 180°, 225°, 270°, and 315°), with the speed of each direction weighed by 0.6, 0.2, 0.6, 1.0, 0.6, 0.2, 0.6, and 1.0, respectively (biased movement in the top left and bottom right directions). No movement action constituted 75% of the time. For each trial, a reward of 1 was given when the agent reached the reward zone (0.4 × 0.4 arbitrary units) located in the center of the arena (1.0 × 1.0 arbitrary units) from a random position outside the reward zone, and no reward was given elsewhere.

For the two-reward-magnitude environment, the reward zone was split into two in the same arena (1.0 × 1.0 arbitrary units), and a reward of 1 was given when the agent reached the right side of the reward zone and a reward of 0.1 was given when the agent reached the left side of the reward zone. For the open-loop environment, the agent was trained first, but it was forced to take a random action in each state regardless of the actor’s computed action probability.

The size of the two-room environment was 2.0 × 4.0 arbitrary units, where a door was located in the middle (0.4 × 0.2 arbitrary units). The agent was trained with A2C for 10^5^ steps, and the same agent was then trained with either the subgoal discovery or control algorithm for direct comparisons. In the subgoal discovery algorithm, the agent started in a random position in the left room and received an intrinsic reward of 1 when it passed the subgoal, defined as the highest DD corresponding to the bottleneck region at the door. The agent also received an external reward of 1 when it reached the reward zone at the bottom right corner (0.4 × 0.4 arbitrary units) of the right room. In the control algorithm, no intrinsic reward was given to the agent but the external reward was adjusted to be 2 at the reward zone.

In the subgoal environment of the object manipulation task, a reward 1 was given when the agent reached a subgoal zone (0.2 × 0.2 arbitrary units) located at the bottom right or top left part of the arena. In each trial, the agent was allowed to visit and collect the intrinsic reward once at the subgoal.

#### A2C algorithm

The A2C (*48*) algorithm was based on the pytorch-a2c-ppo-acktr-gail (https://github.com/ikostrikov/pytorch-a2c-ppo-acktr-gail) and OpenAI baselines (https://github.com/openai/baselines) packages. The actor-critic algorithm is based on the policy gradient and value-based methods. In general, the objective of the policy gradient algorithm is the expected sum of future rewards denoted as *J*(θ) according to

$$J(\theta )=E_{\pi_{\theta }}[\sum_{t=1}^{T}R(s_{t},a_{t})]$$ (18)

where θ is the weight of the neural network, E is expectation, π is a policy, *R*(*s_t_*, *a_t_*) is a reward in a state *s* and action *a* at time *t*, and *T* is a trial end. The actor modifies policy parameters θ by performing gradient ascent to reach the optimum according to the policy gradient theorem

$$\nabla_{\theta }J(\theta )=E_{\pi_{\theta }}[\nabla_{\theta }\text{log}\pi_{\theta }(s,a)\;A^{\pi_{\theta }}(s,a)]$$ (19)

where ∇_θ_ log π_θ_(*s*, *a*) is a score function and *A*^π_θ_^(*s*, *a*) is an advantage function describing the value of a particular action *Q*^π_θ_^(*s*, *a*) relative to the average of all action values in the state [i.e., *V*^π_θ_^(*s*)], defined as

$$\;A^{\pi_{\theta }}(s,a)=Q^{\pi_{\theta }}(s,a)-V^{\pi_{\theta }}(s)$$ (20)

The policy parameter θ is updated by taking a step of gradient ascent according to

$$\theta \leftarrow \theta +\alpha \nabla_{\theta }J(\theta )$$ (21)

where α is a learning rate. In RL, *A*^π_θ_^(*s*, *a*) is unknown and has to be estimated by sampling the TD error δ*_t_* according to the following equation, as δ*_t_* is an unbiased sample of *A*^π_θ_^(*s*, *a*)

$$A^{\pi_{\theta }}(s_{t},a_{t})=E[\delta_{t}]\approx R_{t+1}+\gamma V^{\pi_{\theta }}(s_{t+1})-V^{\pi_{\theta }}(s_{t})$$ (22)

*V*^π_θ_^(*s*) needs to be learned by the critic through supervised regression via function approximation with the neural network according to

$$ℒ(\theta )=\frac{1}{2}\sum {\left\|V^{\pi_{\theta }}(s)-y\right\|}^{2}$$ (23)

where ℒ(θ) is a loss function and *y* is a target state-value, such as a bootstrapped estimate using the previously fitted *V*^π_θ_^(*s*). If the bootstrapped estimate is used as *y*, *V*^π_θ_^(*s*) − *y* becomes *A*^π_θ_^(*s*, *a*). Thus, while the actor in A2C optimizes the agent behavior π_θ_(*s*, *a*), the critic learns to estimate *V*^π_θ_^(*s*).

In the algorithm used in our study, the total loss function was constructed by a weighted sum of the critic loss, actor loss, and entropy terms

$$L_{\text{total}}(\theta )=\beta_{\text{critic}}L_{\text{critic}}(\theta )+\beta_{\text{actor}}L_{\text{actor}}(\theta )+\beta_{\text{entropy}}\text{entropy}$$ (24)

where β_critic_, β_actor_, and β_entropy_ were coefficients for each term. β_actor_ was kept as 1, and β_critic_ and β_entropy_ were changed in the hyperparameter search. Each term was defined as

$$L_{\text{critic}}(\theta )=\sum {\left(A^{\pi_{\theta }}(s,a)\right)}^{2}$$ (25)

$$L_{\text{actor}}(\theta )=-E[\text{log}\pi_{\theta }(s,a)\;A^{\pi_{\theta }}(s,a)]$$ (26)

$$\text{Entropy}=-\sum \pi_{\theta }(s,a)\;\text{log}\pi_{\theta }(s,a)$$ (27)

Different coefficients were assigned to these loss functions, and the total loss was minimized by the gradient descent algorithm.

The following parameters were used when the deep RL agents were trained with A2C: number of training Central Processing Unit (CPU) processes = 16; discount factor γ = 0.95 (0.99 for two-room environment due to its larger arena); learning rate = 10^−4^; RMSprop optimizer ε = 10^−5^; RMSprop optimizer α = 0.99; max norm of gradients = 0.5; number of environment steps to train = 1.6 × 10^3^ for naïve, 1.6 × 10^4^ for intermediate, and 5 × 10^5^ for expert. The generalized advantage estimator with the λ parameter = 0.95 was used to exponentially weigh *n*-step returns (*59*).

To study potential constraints imposed in the mouse cortex, following network architecture and algorithmic hyperparameter space was explored [network architecture: whether actor and critic networks are shared or separated, the number of layers (1, 2, 3, and 4), location of the dropout layer (none, 1, 2, 3, 4, and all), and fraction of dropout (0, 20, and 40%); algorithmic hyperparameter: the coefficient of value-function loss (0, 0.1, and 0.2), coefficient of policy entropy (0, 0.2, and 0.4), and the number of future steps to be considered to estimate *V*^π_θ_^(*s*) (1, 4, and 8)]. We trained a total of 6480 deep RL agents for 1620 hyperparameter configurations (4 agents with random initialization per model).

### Behavior analysis of the deep RL agent

The behavior of the deep RL agent was determined at each learning stage from the initial state by iteratively inputting new states based on previous actions defined by the actor, while parameters for the actor and critic neural network were fixed. In each trial, this procedure was repeated until the agent reached the reward zone (hit) or 300 time steps elapsed (miss). A total of four agents with different seeds for each hyperparameter configuration were trained, and each agent performed 300 trials. Eight performance-optimized agents were trained for the two-reward-magnitude experiment, and 30 performance-optimized agents were trained for the two-room environment. To assess behavior improvement over training, we calculated at each stage the mean correct rate, mean discounted return, median trial duration, and median distance traveled across agents. Agent movement was analyzed similarly to the mouse behavior to determine the state-value function, policy, and subgoal. For the two-reward-magnitude experiment, the reward zone was split into half and a reward of 1 and 0.1 was assigned to the high and low reward side, respectively.

### Activity analysis of the ANN of the deep RL agent

Neural activity in hidden layers of the ANN was determined by measuring outputs of each layer after the activation function for each state of the trajectory. Space tuning of neurons was determined by feeding 100,000 uniformly distributed random state inputs to the network and measuring outputs of each layer after the activation function. The arena was divided into 40 × 40 spatial bins, and activity corresponding to each bin was averaged. This analysis ensured that activity was captured in states that the agent might not visit because of the limited number of trials, which allowed fair comparisons of space representations across different learning stages. Direction tuning of neurons was determined by averaging neural activity corresponding to each direction of the agent movement.

#### Analysis of space and velocity tuning of neurons in the deep RL agent

To quantify space and velocity tuning of individual neurons in the deep RL, spatial coherence, border index, and whether a neuron encodes velocity were determined. Spatial coherence was calculated by Pearson’s correlation coefficient between activity of each spatial bin and mean activity of eight nearest bins. Border index was calculated by the difference between the maximal length of any of four borders touching a spatial activity field of a neuron and the mean distance of the activity field to the nearest border, divided by the sum of the two values (*60*). The activity field was determined by thresholding at *z* score of spatially binned activity above 1. To determine whether a given neuron encodes velocity, we first performed one-way ANOVA to statistically (*P* < 0.05) identify its preferred direction. The direction-tuned neuron was then statistically (*P* < 0.05) tested for speed modulation by computing Pearson’s correlation coefficient between the binned speed and neural activity at the preferred direction.

#### Analysis of the state-value function and subgoal representation in the ANN of the deep RL agent

To test whether the space tuning of each neuron in the ANN of the deep RL agent corresponded to the state-value or subgoal representations, we calculated Pearson’s correlation coefficient between the space tuning and state-value function or subgoal. We shuffled the spatial bins of agent trajectories 1000 times and computed shuffled space tuning for each neuron. *P* values were then computed with Pearson’s correlation coefficient between the shuffled space tuning and state-value function or subgoal. We considered a neuron deemed to be representing the state-value or subgoal when the *P* value was less than 0.05 with the Benjamini-Hochberg false discovery rate.

For the two-reward-magnitude experiment, it was determined whether the peak location of space tuning was on the right (high reward) side or left (low reward) side of the arena. The enrichment index was then calculated according to Eq. 16. As a control, the enrichment index was also determined for the bottom side versus the top side. As the deep RL agent more frequently reached the right (high reward) side of the reward zone from the bottom side of the arena, the enrichment index for the control was slightly positive. For the open-loop experiment, the fraction of state-value–representing neurons was determined for closed-loop and open-loop environments, and the normalized change was calculated according to Eq. 17.

For the two-room environment, the arena (2.0 × 4.0 arbitrary units) was binned to 40 × 80 to obtain space tuning of a neuron. The location of space tuning was then determined for each neuron, and if it resides in the door region (19 to 22 bins for *x*, 37 to 44 bins for *y*), the neuron was deemed to be representing a subgoal. The fractions of subgoal neurons were determined among active neurons for the subgoal discovery and control algorithms, and enrichment difference index was determined as

$$\text{Enrinchment difference index}=\frac{{\text{Fraction of neurons}}_{\text{subgoal discovery}}-{\text{Fraction of neurons}}_{\text{control}}}{{\text{Fraction of neurons}}_{\text{subgoal discovery}}+{\text{Fraction of neurons}}_{\text{control}}}$$ (28)

To determine whether the distribution of the space tuning in the task environment was skewed toward a quadrant with the intrinsic reward, the peak location of space tuning was determined for each neuron. The representation enrichment was measured as the fraction of neurons residing in the quadrant of interest among summed fractions over all the quadrants.

#### Analysis of the policy representation in the ANN of the deep RL agent

To study policy representations of neurons in the ANN of the deep RL agent, space and direction tuning of each neuron were considered. The state of interest was first determined by looking at the top 5% of the most active spatial bins in the space tuning. We then determined the movement direction distribution of the agent in these spatial bins by averaging movement distributions and weighing them by the normalized activity level in each bin. Pearson’s correlation coefficient was then calculated between the direction tuning of each neuron and the resulting direction distribution of the agent. *P* values were obtained by shuffling the agent movement direction 1000 times, computing shuffled direction tuning, and calculating Pearson’s correlation coefficient between the shuffled direction tuning of each neuron and the agent’s direction distribution. Neurons with *P* value of less than 0.05 with the Benjamini-Hochberg false discovery rate were considered to be policy-representing cells. For the open-loop experiment, the fraction of policy-representing neurons was determined for closed-loop and open-loop environments, and the normalized change was calculated according to Eq. 17.

#### Analysis of the sparse representation in the ANN of the deep RL agent

Fractions of active cells were determined on the basis of whether 100,000 uniformly distributed random state inputs caused nonzero activity in each neuron. This analysis was necessary to make fair comparisons of space representations across different learning stages, as the agent’s trajectories were very different. If these inputs led to nonzero activity in any spatial bins in a given neuron, we defined it as an active cell, and we defined it as an inactive cell otherwise.

To visualize the learning-dependent selection process of active neurons in the deep RL agent, active neurons at either intermediate or expert stages were embedded in the *t*-distributed stochastic neighbor embedding (*t*-SNE) coordinate based on their space or direction tuning at the intermediate stage.

#### Model selection of the task-performance-optimized deep RL agent

Extensive hyperparameter search revealed that the task performance was correlated with abundance of the state-value- and policy-encoding cells. To further investigate the other properties of the ANN, we focused on one task-performance-optimized deep RL model based on three features in each neural network: the fraction of state-value neurons, fraction of policy neurons, and sparse coding. For each feature, the value was normalized so that it is bounded between 0 (lowest among all the RL agents) and 1 (highest among all the RL agents). These three normalized values were then summed, and the resulting number was used as a metric to rank the deep RL agents.

### Neural inactivation in the ANN of the deep RL agent

To investigate the effect of neural inactivation on agent movement, activity of either 20, 40, 60, 80, or 100% of state-value– or policy-representing neurons in the hidden layer was set to be 0. Given fully learned and fixed parameters for the neural network, agent trajectories and rewards were determined for 300 trials for each agent.

### Statistics

Because of the variable number of neurons and cortical regions sampled in each mouse, fractions of task-related, state-value, policy, and subgoal neurons and normalized changes in fractions of state-value and policy neurons between the closed- and open-loop configurations were displayed with mean ± SD obtained from randomly sampled mice with replacement over 1000 shuffles to estimate the variability. Other figures were displayed with mean ± SEM unless stated otherwise.

## Notes

View/request a protocol for this paper from *Bio-protocol*.
