# Supplementary material for: Representation learning in the artificial and biological neural networks underlying sensorimotor integration
Source: Sci Adv. 2022 Jun 3;8(22):eabn0984. doi: 10.1126/sciadv.abn0984 (PMC9166289; doi:10.1126/sciadv.abn0984)
Supplement: Supplementary file 1 — Figs. S1 to S7 [file sciadv.abn0984_sm.pdf]

Science Advances
AAAS

Supplementary Materials for

Representation learning in the artificial and biological neural networks underlying sensorimotor integration

Ahmad Suhaimi *et al.*

Corresponding author: Hiroshi Makino, hmakino@ntu.edu.sg

Sci. Adv. **8**, eabn0984 (2022)
DOI: 10.1126/sciadv.abn0984

This PDF file includes:

Figs. S1 to S7

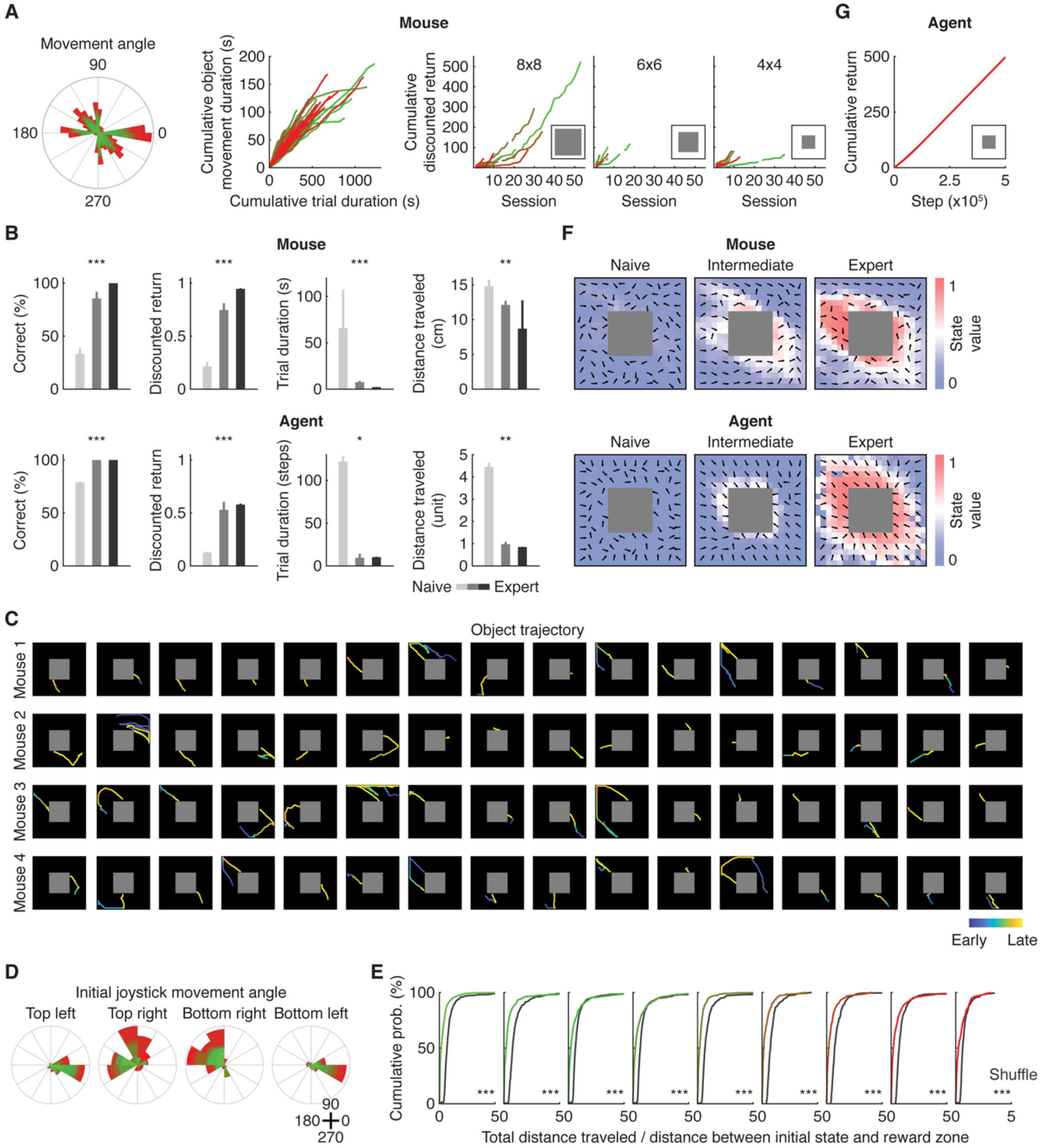

Fig. S1. Behavior, state-value function and policy of the mouse and deep RL agent.

(A) Left. Angle distribution of the object movement manipulated by individual expert mice (n = 9 mice), indicated by different colors. Note that the object movement was biased in the diagonal directions (top left and bottom right) due to the relative position of the right paw to the joystick, and cardinal directions due to the edges of the arena. Middle. Cumulative object movement duration as a function of cumulative trial duration of individual expert mice. The slopes indicate fractions of the time spent by mice moving the object. Right. Learning curves at different training stages. Grey box in the arena indicates a reward zone at each training phase.

(B) Correct rate, discounted return, median trial duration and median distance traveled in each trial of mice (top) (correct rate: ***$p < 0.001$, discounted return: ***$p < 0.001$, one-way ANOVA, trial duration:

***p < 0.001, distance traveled: **p < 0.01, Kruskal-Wallis test, n = 6, 7 and 9 mice for naïve, intermediate and expert) and deep RL agents (bottom) (correct rate: ***p < 0.001, discounted return: ***p < 0.001, one-way ANOVA, trial duration: ***p < 0.001, distance traveled: **p < 0.01, Kruskal-Wallis test, n = 4 agents for naïve, intermediate and expert).

(C) Example object trajectories of 16 consecutive trials from four expert mice. Color represents time. Note that the starting position was randomized in each trial.

(D) Angle distribution of initial joystick movement at the trial onset sorted by initial object position (top left, top right, bottom right and bottom left). Different colors indicate individual expert mice (n = 9 mice).

(E) Cumulative probability distributions of the total distance traveled divided by the initial distance to the reward zone. Comparisons were made against object trajectories derived from shuffled joystick movements (***p < 0.001, Kolmogorov-Smirnov test). Different colors indicate individual expert mice (n = 9 mice).

(F) Examples of the state-value function and policy at different stages of learning in mice (top) and deep RL agents (bottom).

(G) Learning curves for different deep RL agents (n = 4 agents), indicated by different colors. The lines are overlapping.

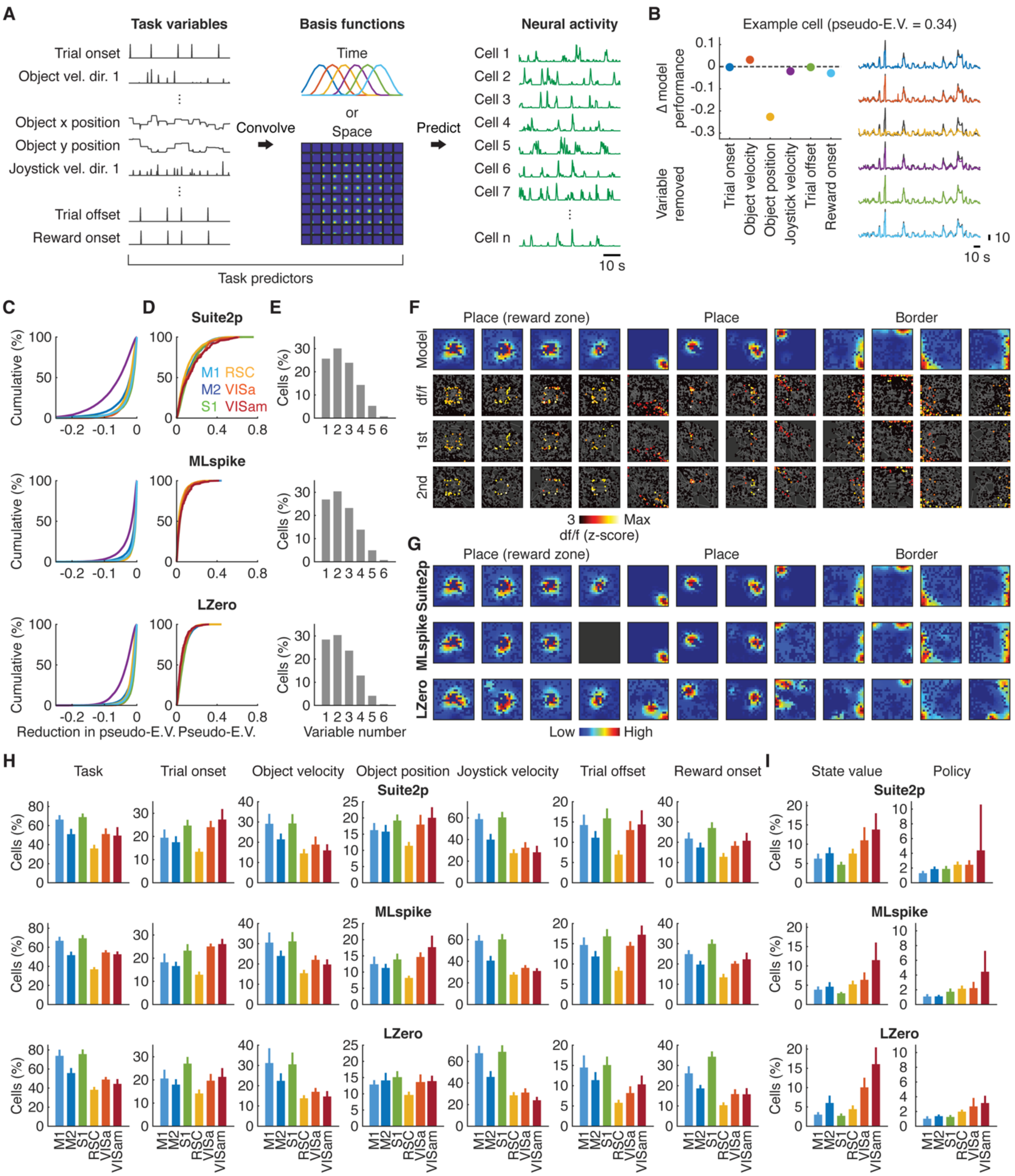

Fig. S2. Generalized linear model (GLM) with different deconvolution methods for neural representations in the mouse cortex.

(A) Schematic of GLM. Task variables were convolved with basis functions in time or space and the model was trained to fit the deconvolved calcium signal for each neuron by regression.

- **(B)** Example neuron encoding the object position. Left. The model performance, assessed by pseudo-explained variance (E.V.), decreased when the object position was not included in the model. Right. Actual (black) and predicted (colored) activity when each task variable was removed.
- **(C)** Cumulative probability distribution of reduction in pseudo-E.V. for each task variable computed by Suite2p (top), MLspike (middle) or LZero (bottom). The color scheme is the same as (B).
- **(D)** Cumulative probability distribution of pseudo-E.V. for each cortical region computed by Suite2p (top), MLspike (middle) or LZero (bottom).
- **(E)** Probability distribution of neurons encoding task variables. Note that the majority of the neurons showed mixed selectivity (≥ 2 task variables).
- **(F)** Examples of space tuning derived either from GLM (1st row), deconvolved df/f (2nd row), deconvolved df/f from the first half of the session (3rd row) and deconvolved df/f from the second half of the session (4th row) using Suite2p.
- **(G)** Comparisons of space tuning of the same neurons in each column modeled by GLM using Suite2p (top), MLspike (middle) or LZero (bottom). Dark box indicates a neuron with no activity.
- **(H)** Distribution of each task-variable-encoding neurons across different cortical regions computed with Suite2p (top), MLspike (middle) or LZero (bottom).
- **(I)** Distribution of state-value- or policy-encoding neurons across different cortical regions computed with Suite2p (top), MLspike (middle) or LZero (bottom).

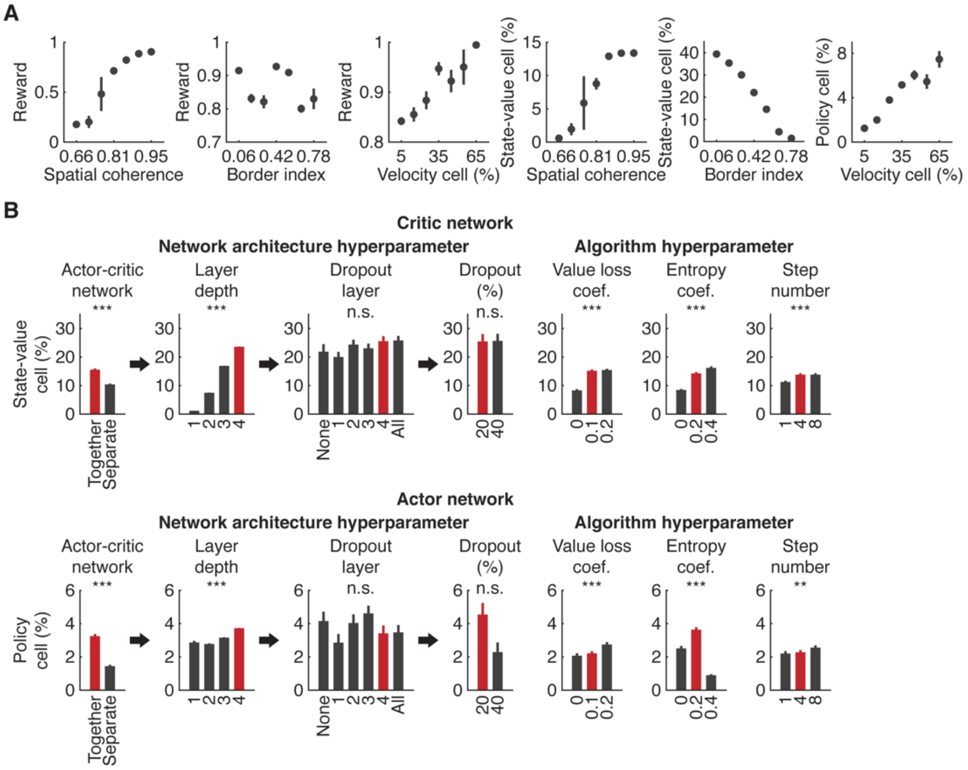

Fig. S3. Exploration of the hyperparameter space for the tuning properties, state-value and policy.

(A) Dependence of the reward, fraction of state-value- or policy-encoding neurons on space or velocity-encoding properties in the deep RL agent.

(B) Deep RL agent model selection by hyperparameter search. Red hyperparameters were selected as the task-performance-optimized agent. Critic: actor-critic network: ***$p < 0.001$, Wilcoxon rank-sum test, $n = 810$, 810 agents (from left to right); layer depth: ***$p < 0.001$, Kruskal-Wallis test, $n = 81$, 189, 243, 297 agents; dropout layer: $p = 0.17$, Kruskal-Wallis test, $n = 27$, 54, 54, 54, 54, 54 agents; dropout (%): $p = 0.99$, Kruskal-Wallis test, $n = 27$, 27 agents; value-loss coefficient: ***$p < 0.001$, Kruskal-Wallis test, $n = 540$, 540, 540 agents; entropy coefficient: ***$p < 0.001$, Kruskal-Wallis test, $n = 540$, 540, 540 agents; step number: ***$p < 0.001$, Kruskal-Wallis test, $n = 540$, 540, 540 agents. With Bonferroni correction. Actor: actor-critic network: ***$p < 0.001$, Wilcoxon rank-sum test, $n = 810$, 810 agents (from left to right); layer depth: ***$p < 0.001$, Kruskal-Wallis test, $n = 81$, 189, 243, 297 agents; dropout layer: $p > 0.05$, Kruskal-Wallis test, $n = 27$, 54, 54, 54, 54, 54 agents; dropout (%): $p > 0.05$, Kruskal-Wallis test, $n = 27$, 27 agents; value-loss coefficient: ***$p < 0.001$, Kruskal-Wallis test, $n = 540$, 540, 540 agents; entropy coefficient: ***$p < 0.001$, Kruskal-Wallis test, $n = 540$, 540, 540 agents; step number: **$p < 0.01$, Kruskal-Wallis test, $n = 540$, 540, 540 agents. With Bonferroni correction.

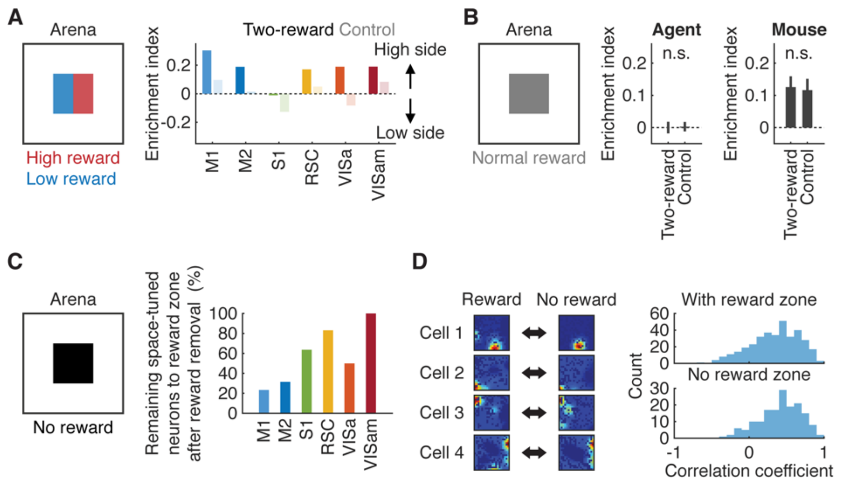

Fig. S4. Further analysis of the two-reward-magnitude and interleaved-reward experiments.

(A) Two-reward-magnitude experiment. Left. Enrichment index for the high/low reward side comparison (e.g. right/left sides) or control side comparison (e.g. bottom/top sides) in different cortical regions.

(B) No difference in the enrichment index in the deep RL agent and mouse when the reward is equally distributed in the reward zone (agent: $p = 0.92$, t-test, $n = 4$ agents; mouse: $p = 0.69$, t-test, $n = 9$ mice).

(C) Interleaved-reward experiment. Remaining fraction of neurons tuned to the reward zone after reward removal. Note that the reduction is less prominent in the posterior regions of the cortex.

(D) Left. Space tuning of the same neurons in rewarded and non-rewarded trials within the same session. Individual neurons were fitted to independent GLMs for each condition. Right. Histogram showing Pearson's correlation coefficient between space tuning of the same neurons. Neurons spatially tuned to the reward zone were either included (top) or excluded (bottom).

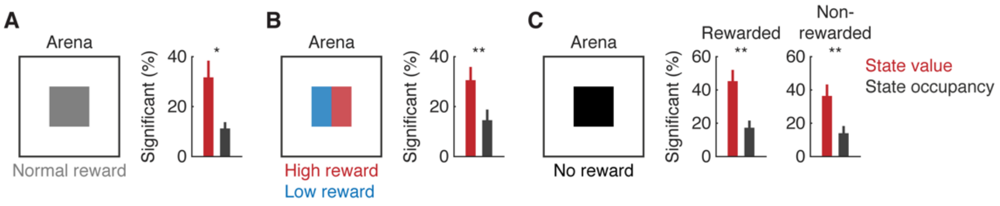

Fig. S5. Space tuning represents the state value but not state occupancy.

(A) Higher fraction of space-tuned neurons representing the state value than state occupancy (*$p < 0.05$, n = 6 mice, bootstrap, naïve stage).

(B) Same as (A) for the two-reward-magnitude experiment (**$p < 0.01$, n = 6 mice, bootstrap).

(C) Same as (A) for the interleaved-reward experiment with or without reward trials (**$p < 0.01$, n = 5 mice, bootstrap).

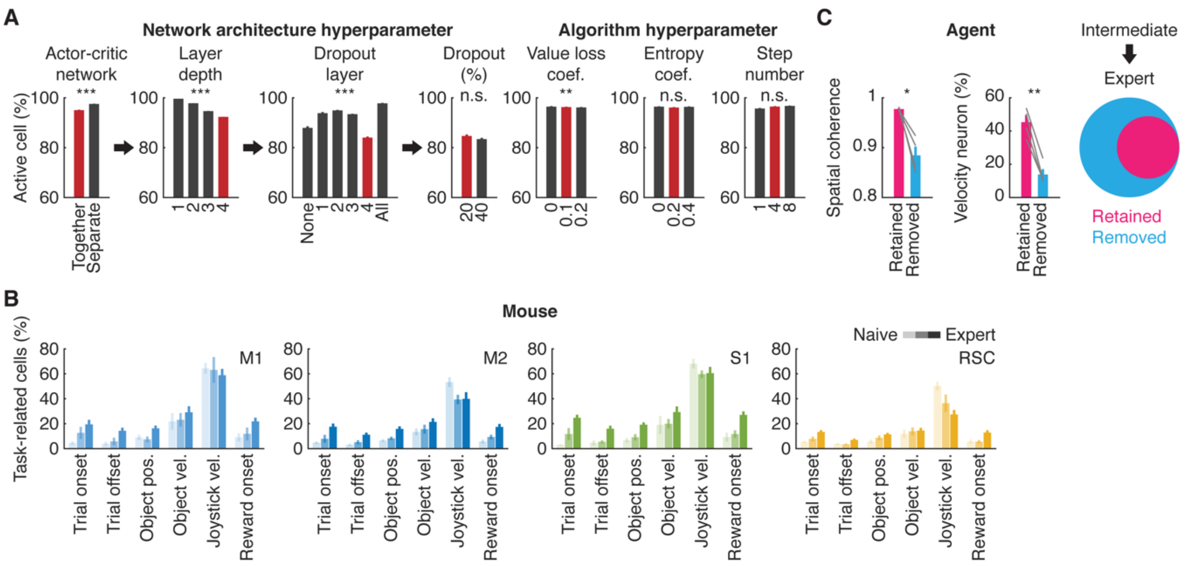

Fig. S6. Exploration of the hyperparameter space for sparse coding.

(A) Deep RL agent model selection by hyperparameter search. Red hyperparameters were selected as the task performance-optimized agent. Actor-critic network: ***$p < 0.001$, Wilcoxon rank-sum test, n = 810, 810 agents (from left to right); layer depth: ***$p < 0.001$, Kruskal-Wallis test, n = 81, 189, 243, 297 agents; dropout layer: ***$p < 0.001$, Kruskal-Wallis test, n = 27, 54, 54, 54, 54, 54 agents; dropout (%): $p = 0.06$, Kruskal-Wallis test, n = 27, 27 agents; value-loss coefficient: **$p < 0.01$, Kruskal-Wallis test, n = 540, 540, 540 agents; entropy coefficient: $p = 0.54$, Kruskal-Wallis test, n = 540, 540, 540 agents; step number: $p > 0.05$, Kruskal-Wallis test, n = 540, 540, 540 agents. With Bonferroni correction.

(B) Learning-dependent changes in task-related cell fractions in each cortical region.

(C) Learning-dependent selective retainment of spatially coherent and velocity-tuned neurons in the deep RL agent (*$p < 0.05$, **$p < 0.01$, t-test, n = 4 agents).

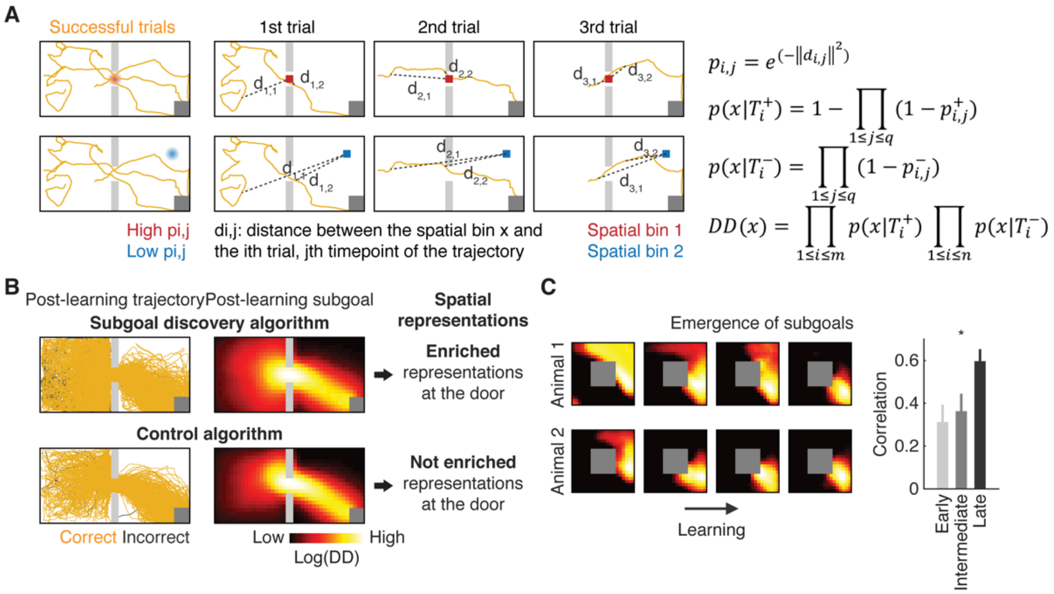

Fig. S7. Subgoal identification with diverse density.

(A) Subgoal identification. A subgoal was determined by computing log diverse density (DD) of successful and unsuccessful trials. The most diversely dense region is the spatial bin where the agent passes through on multiple successful trajectories and not on unsuccessful ones. The distance between each spatial bin x of the arena and each point j of the 10-ms binned trajectory in each trial i was used as approximation to the relative probability that given a spatial bin x corresponds to a subgoal. q is the number of 10 ms bins, and m and n are the numbers of successful (+) or unsuccessful (-) trials, respectively.

(B) The subgoal discovery algorithm and control algorithm yield similar trajectories and log(DD) distribution in the arena but result in different enrichment in space-tuned neurons at the door.

(C) Left. Examples of learning-dependent convergence in log(DD). Right. Learning-dependent increase in the correlation of log(DD) relative to the last session (*$p < 0.05$, one-way ANOVA, n = 9 mice).
